# Ionic Liquid-Based Electrolytes for Energy Storage Devices: A Brief Review on Their Limits and Applications

**DOI:** 10.3390/polym12040918

**Published:** 2020-04-15

**Authors:** K Karuppasamy, Jayaraman Theerthagiri, Dhanasekaran Vikraman, Chang-Joo Yim, Sajjad Hussain, Ramakant Sharma, Thandavaryan Maiyalagan, Jiaqian Qin, Hyun-Seok Kim

**Affiliations:** 1 Division of Electronics and Electrical Engineering, Dongguk University-Seoul, Seoul 04620, Korea; karuppasamyiitb@gmail.com (K.K.); v.j.dhanasekaran@gmail.com (D.V.); akreal@naver.com (C.-J.Y.); 2 Centre of Excellence for Energy Research, Centre for Nanoscience and Nanotechnology, Sathyabama Institute of Science and Technology (Deemed to be University), Chennai 600119, India; j.theerthagiri@gmail.com; 3 Graphene Research Institute, Sejong University, Seoul 05006, Korea; shussainawan@gmail.com; 4 Institute of Nano and Advanced Materials Engineering, Sejong University, Seoul 05006, Korea; 5 Integrated Organic Electronics Lab, School of Electrical Engineering, Korea Advanced Institute of Science and Technology, Daejeon 34141, Korea; fergussonian.ramakant@gmail.com; 6 Electrochemical Energy Laboratory, Department of Chemistry, SRM Institute of Science and Technology, Kattankulathur 603203, India; maiyalagan@gmail.com; 7 Research Unit of Advanced Materials for Energy Storage, Metallurgy and Materials Science Research Institute, Chulalongkorn University, Bangkok 10330, Thailand; jiaqian.q@chula.ac.th

**Keywords:** ionogel, polymer electrolytes, energy storage, electric double-layer capacitors, interfacial property

## Abstract

Since the ability of ionic liquid (IL) was demonstrated to act as a solvent or an electrolyte, IL-based electrolytes have been widely used as a potential candidate for renewable energy storage devices, like lithium ion batteries (LIBs) and supercapacitors (SCs). In this review, we aimed to present the state-of-the-art of IL-based electrolytes electrochemical, cycling, and physicochemical properties, which are crucial for LIBs and SCs. ILs can also be regarded as designer solvents to replace the more flammable organic carbonates and improve the green credentials and performance of energy storage devices, especially LIBs and SCs. This review affords an outline of the progress of ILs in energy-related applications and provides essential ideas on the emerging challenges and openings that may motivate the scientific communities to move towards IL-based energy devices. Finally, the challenges in design of the new type of ILs structures for energy and environmental applications are also highlighted.

## 1. Introduction

In the recent times, most of the transportable smart devices and some of the hybrid electric vehicles, which are marketed to present day customers, are equipped with the light weight electrochemical energy storage (EES) devices, include lithium-ion batteries [1,2,3,4] (LIBs) and supercapacitors [5,6,7,8] (SCs), which is the backbone of commercially available portable electronic devices [9,10]. However, to date, uses of LIBs and SCs as a power sources for large scale applications, including electric vehicles, are a distant reality. Operational safety is one of the vital reasons hindering their use for large scale application; hence, their widespread uses need to be addressed. In addition, the transport property and structural stability of the ionic species are extremely crucial and responsible for the efficient outputs in energy storage devices. In the light of this fact, high current (i.e., automotive applications) operating devices are required to have storage devices that have higher power density and faster ion transport properties. Previous studies have suggested that one of the most favorable approaches to simultaneously progress the safety, along with energy and power densities, is the incorporation of ILs into the electrolyte system [11].

In past decades, room temperature ionic liquids (RTILs) have been acknowledged with noteworthy attention due to their excellent miscellaneous properties, such as thermal and chemical stability, tunable structure over the wide range of operating temperature, a broad electrochemical stability window, high ionic conductivity in the range of 10^−^^3^–10^−^^2^ S cm^−1^ at room temperature, and non-flammability as a potential candidate for EES devices, such as LIBs [12], electric double-layer SCs [13,14,15], proton exchange membrane fuel cells, and solar cells [16,17,18,19]. Using ILs as an alternative to organic electrolytes has the advantage of improving the ions’ mobility, as well as eliminating the hazards associated within the organic electrolytes. Additionally, ILs often have comparable zero or negligible vapor pressure at normal temperatures due to their high thermal stability. Because of the above statements, ILs are widely used as solvents or electrolytes for energy storage applications in recent times [7,18,20,21,22,23,24,25,26,27].

Typically, ILs are organic salts, also defined as molten salts, which have a lower melting point (<100 °C) with a wide degree of variation. Moreover, they are comprised of organic cations, such as an pyridinium (PY) [28], imidazolium (Im) [29,30,31,32], pyrrolidinium (PYR) [33,34,35,36], ammonium [37], and sulfonium [38], derivatives joined inorganic or organic anions, such as BF_4_^−^ [39,40], PF_6_^−^ [30], triflate (CF_3_SO_3_^−^)[41], and bis(trifluoromethanesulfonyl imide) (TFSI) ((CF_3_SO_2_)_2_N^−^) [42,43]. Thus, the different combinations of cations and anions [44,45,46,47] unveil the numerous possibilities to architect the ILs with capable compounds for the desired applications.

**Figure 1:**
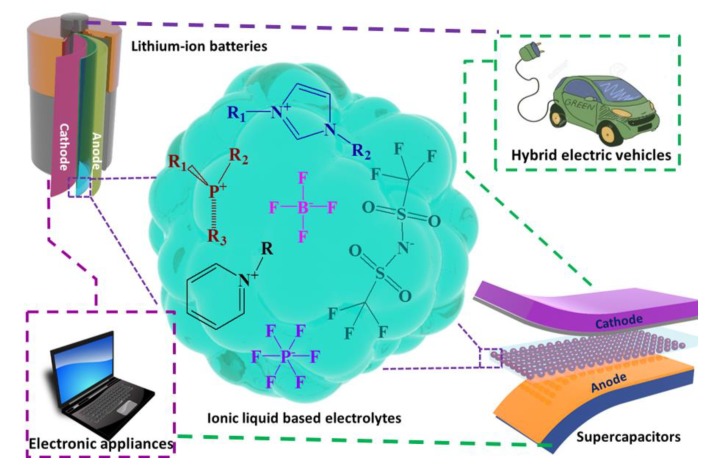
Schematic representation of ionic liquid (IL)-based electrolytes applications in energy storage devices (lithium ion batteries (LIBs) and supercapacitors (SCs)).

In this review, we aimed to present the recent developments of IL-based electrolytes for their potential applications in LIBs and SCs. Additionally, some of the strategies, opportunities, and challenges are discussed for the more rational design of ILs structures to make it more suitable for fabricating green EES devices. Especially, discussions are focused to cover-up the outstanding physicochemical and electrochemical properties of some potentially modified electrolytes with ILs for their applications in energy storage systems. However, to keep this review short, precise, and useful to present-day researchers, this review assessed, examined, and discussed articles published after 2010; therefore, some of the excellent research works prior to 2010 are not cited. To avoid plausible distractions, the content of this review article is divided into two different broad categories, as follows: (a) IL-based electrolytes for LIB applications and (b) IL-based electrolytes for SC applications. A schematic representation of IL-based electrolytes employed in LIBs and SCs is denoted in Figure 1.

## 2. IL-Based Electrolytes for LIBs Application

It is well known that the specific energy densities of LIBs are quite high compared to other conventional batteries and SCs, but it certainly needs improvement in terms of power density. To improve LIBs power density, LIBs components, such as anode, cathode, and electrolytes, are deeply investigated and various replacements have been reported elsewhere [48,49]. To date, various electrolytes have been prepared and employed in this aspect. Moreover, this part of review is focused on the IL-based liquid, gel, and solid polymer electrolytes prepared by various techniques/activation processes for LIB applications.

In general, the LIB mainly consists of three components: (1) the positive electrode, which is typically based on metal oxides and phosphates; (2) the negative electrode, which is generally graphite or metallic lithium; and (3) an electrolyte, which can be either in a solid, liquid, or gel form. Electrolytes facilitate the migration of ionic charge carriers within the LIBs and are hence considered to be the heart of LIBs [50,51]. To be a good electrolyte, certain characteristics in nature are necessary in the electrolyte comprised LIBs for stable and safe operation. The important functions of electrolytes in LIBs are as follows: (a) the rapid migration properties during the charge-discharge process between the two electrodes, which is essential to transport of lithium ions; (b) robustness of electrochemical and chemical properties; (c) the high boiling point and low melting point, which are crucial for broad region of operating temperatures; (d) negligible vapor pressure at room temperature, which reduces the flammability of the electrolyte. Although different types of electrolytes have been designed and employed in LIBs, the two most widely accepted are liquid and polymer electrolytes (which can be further categorized into solid, gel, and composite electrolytes).

### 2.1. Organic Carbonates and Ionic Liquid-Based Binary Liquid Electrolytes

In general, during battery operations, the electrolytes of organic solvents which have inflammable properties may imitate the fire or explosions owing to the abominable conditions or short circuit. Thus, operational safety issues must be addressed before it could be widely launched for practical applications. To advance operational safety, ILs have been incorporated with an organic carbonate-based electrolyte that has efficiently enhanced the thermal stability of lithium cells [52]. In addition, IL has better conductivity compared to conventional organic electrolytes, and it could be overwhelmed by the inclusion of selective carbonates in the ILs, ensuing the development of low viscous mixtures and thereby further improving the conductivity and thermal stability [53]. From this perspective, ILs have been broadly considered and are regarded as one of the secured electrolytes for LIB applications because of their excellent properties, such as high ionic conductivity, negligible vapor pressure, low flammability, and high thermal stability. In addition, maximum ILs have better ionic conductivity and considerable electrochemical stability; hence, they are outstanding alternatives to flammable organic carbonate solvents. Furthermore, the choice of the anions and cations in ILs is very important, and it plays a vital role in evaluating the viscosity and solubility of ILs [37,43].

Among the different families of ILs, the ILs containing TFSI anions and PYR cations have been widely studied and used for LIB applications, owing to their excellent electrochemical and thermal stabilities [33,42,54,55]. Between the TFSI anionic ILs, 1-butyl-3-methylpyrrolidinium bis(trifluoromethanesulfonyl)imide (PYR_14_TFSI) is of particular interest because of its excellent air and thermal stabilities (up to 300 °C). It also exhibits a cathodic stability more than 5.5 V, which makes it a potential electrolyte candidate for LIBs [52,56]. Kuhnel et al. prepared and investigated a ternary electrolyte mixture that contains PYR_14_TFSI and lithium bis(trifluoromethanesulfonyl)imide (LiTFSI), along with a propylene carbonate (PC) solvent [52]. Interestingly, it was observed that the properties can be tuned by adjusting the amount of IL in electrolyte mixtures. It was found that the mixture containing 80 wt% IL has non-flammable properties, with promising cycling performance, at room temperature and 60 °C, as well [52]. Likewise, the flammability and thermal stability of the electrolyte solutions of 1-butyl-1-methyl pyrrolidinium bis(trifluoromethanesulfonyl)imide (PYR_14_TFSI), fluoroethylene carbonate (FEC), ethylene carbonate (EC), and vinylene carbonate (VC) were thoroughly investigated by Ye et al. [57]. They used the carbonate solvents as solid electrolyte interphase (SEI)-forming additives that can make a firm SEI layer over the electrode and protect the electrode from the reductive decomposition of the IL. From their experiments, they observed that the inflammability and capacity declining of the electrolytes were found to be enormously decreased due to the addition of VC along with the IL. Hofmann et al. [58] investigated the behaviors of poly(vinylidene fluoride) hexafluoropropylene (PVdF-co-HFP) polymer electrolytes based on ILs (PYR_13_), lithium bis(trifluoromethylsulfonyl)azanide, and organic carbonates as electrolyte components. Interestingly, the maximum discharge capacity achieved at 100 mA h g^−1^ for a graphite/IL-EC/PC electrolyte/lithium manganese nickel cobalt oxide (LiMNC) cell [58]. In addition, ILs, such as PYR_14_TFSI, 1-methoxyethoxymethyl(tri-*n*-butyl)phosphonium bis(trifluoromethanesulfonyl)amide (MEMBu_3_PTFSI), N-propyl-n-methylphosphonium bis((trifluoromethanesulfonyl imide (PP_13_TFSI), and organic carbonates electrolytes, has demonstrated strong combined effect which caused the high reversibility of lithium ion intercalation, as well as reduction of SEI formation over the electrode-electrolyte interfaces [2,59].

**Figure 2:**
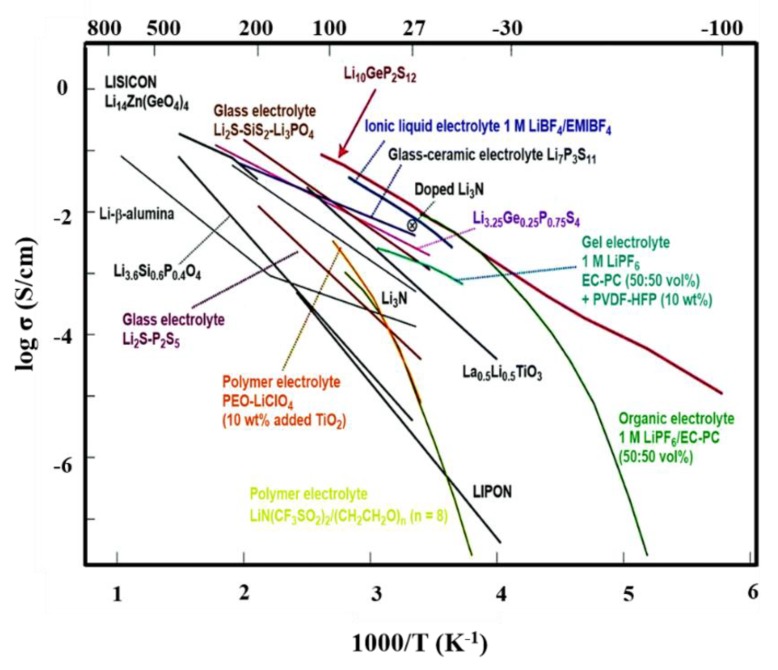
Comparison of ionic conductivities of different types of electrolytes for LIBs [71]. Copyright 2016 The Royal Society of Chemistry.

The inclusion of ILs in organic carbonates have reduced the electrochemical stability window profoundly compared to pure ILs, which is proved in the lot of related literatures [36,60,61,62,63]. In the meantime, the compatibility between the electrolyte and electrode leads to improve the anodic stability greatly. For instance, the electrolyte 0.5 M lithium nitrate (LiNO_3_) in PC-pyrrolidinium nitrate (PYRNO_3_) exhibited good conductivity and an electrochemical stability window that is great enough to assure the safe de-insertion and insertion of lithium into LiFePO_4_ [64,65,66,67]. A new type of organic liquid electrolyte, consisting of Pip_13_TFSI and dimethoxyethane (DME), was studied and reported their improved cell specific capacity and cycle life in lithium-metal batteries [68,69,70]. The comparison diagram of conducting properties of different types of electrolytes used for LIBs [71,72,73,74,75] is shown in Figure 2. In the conductivity plot of LIBs, the conductivity of pristine ILs are three orders better than conventional organic liquid electrolyte and gel polymer electrolytes, which is due to better ions-mobility of ILs. Further, it acts as a co-solvent during the preparation of electrolytes that avoid the solvent effect in electrolyte films.

**Figure 3:**
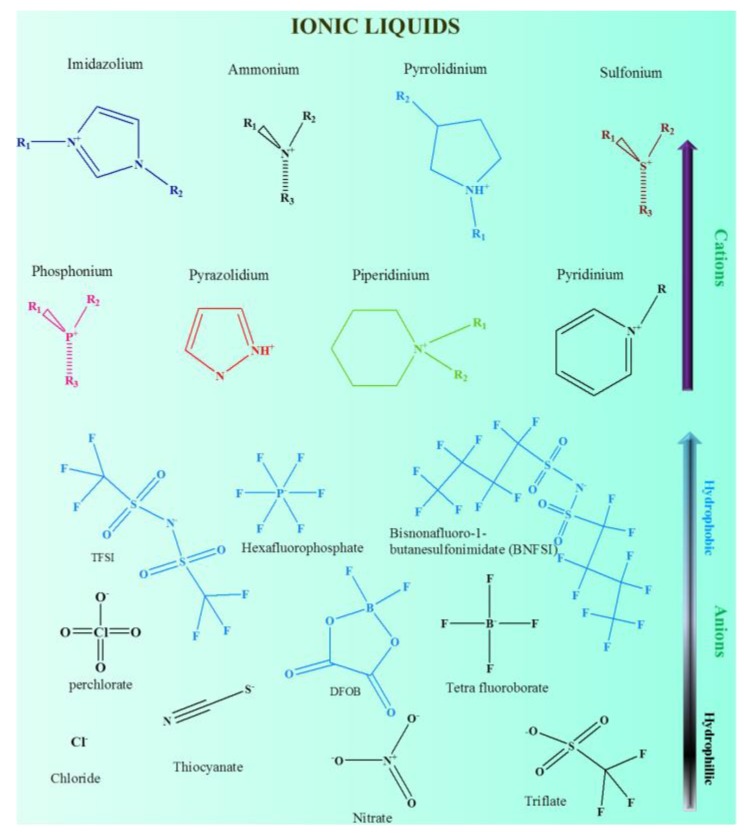
Cations and anions structures of some important ILs.

Depending on the ionic structure, ILs can be either protic or aprotic. Due to enormous available cations and anions structure, different combinations of ILs were studied. Figure 3 shows the cations and anions structure of some important ILs for energy storage systems that are discussed in this review. As we know, the bigger the size of the anion, the weaker the coordination between cation and anion, which in turn facilitates the dissociation of ions very easily in the solvent medium. Recent reports have revealed that the IL electrolytes with bis(fluorosulfonyl imide) (FSI) anions (FSI^−^) exhibit higher ionic conductivities and lower viscosities, which are due to the lesser dimensional size of the FSI^−^ compared to the TFSI anion [42,73]. In some circumstances, the ILs, together with a carbonate solvent, showed a higher viscosity and lower conductivity, which resulted in poor cycling behaviors than that of the organic electrolytes (LiPF_6_ in organic carbonates), particularly at high-rate cycling performances. This is due to the following factors: (i) the formation of an inaccurate random SEI layer on the negative electrodes [76] and (ii) sluggish migration of ions and cumbersome absorption of porous electrodes [77].

Liao et al. [76] reported the prime application of sulfolane (SL), mixtures of SL with the conducting salt LiClO_4_ and carbonates (DMC, PC) in LIB cells. Yet, an ultra-quick capacity fading was detected in graphite anode compartment. Li et al. evidenced the feasibility of sulfites and SL blend as electrolyte solvents with conducting salt of lithium bis(oxalato) borate (LiBOB), which results in the reduction of the flashpoints significantly for practical application of LIBs [78]. To overcome the aforesaid issues, most research has focused on identifying new ILs with negligible vapor pressures and high flash points for LIBs [54,79,80,81,82].

Recently, 1-allyl-3-methylimidazolium (AMIm)TFSI and 1-methyl-3-propylimidazolium (Im_13_)TFSI-based electrolytes with PC were compared by Wang et al. and revealed the formation of (in an 1-allyl-3-methylimidazolium bis(trifluoromethanesulfonyl)imide (AMImTFSI)-based electrolyte) a firm protective layer over the electrode surface, which supports subduing the decomposition of solvent and increasing the cycling behavior of the LIBs [83]. They reported that the Li/LiFePO_4_ coin cell delivered the stable discharge capacities of ~151 mA h g^−1^ after 100 cycles at 0.1 C rate, with a retention of 97.4%. Similar behavior was exhibited for PYR_14_TFSI, as well as AMImTFSI using graphite and lithium manganese nickel cobalt oxide (LMNC) as the anode and cathode, respectively [43,52,84,85].

With the intention of enhancing the safety and performance of LIBs at elevated temperatures, the properties of hybrid organic electrolytes (i.e., ILs in organic carbonates), such as their flammability and volatility, can be measured and repressed through the careful modification of organic solvent content [35,86,87]. The hybrid organic electrolytes containing PYR_13_TFSI:LiTFSI:(EC/diethyl carbonate (DEC) 1:1 mol%) (60:10:30 mol%) were performed as a non-flammable electrolyte at ambient temperature and showed a similar performance to marketable liquid electrolytes in both Li/Li_4_Ti_5_O_12_ (lithium titanate (LTO)) and Li/LiFePO_4_ (LFP) half-cells [35]. An analogous type of 0.3 M LiTFSI in PYR_13_TFSI: vinylene carbonate (VC):(EC/DMC 1:1 wt%) (65:5:30 vol%) electrolyte delivered the highest discharge capacity of 150 mA h g^−1^ at 1 C rate for a Li/LiFePO_4_ half-cell, at 75 °C [86]. Some important electrochemical and cycling properties of the electrolytes containing both ILs and carbonate solvents are charted in Table 1.

Recently, Khalid et al. [88] developed a new category of hybrid IL/organic electrolyte for a high-temperature application using 1-methyl-1-propylpiperidinium bis(trifluoromethanesulfonyl)-imide (Pip_12_TFSI)-based liquid electrolytes. They employed three-dimensional nano-silicon electrodes as a working electrode with the metallic lithium as a reference cum counter electrode. It exhibited the highest specific capacities of 1912 mA h g^−1^ and 2230 mA h g^−1^ at 0.41 and 0.52 mA h cm^−2^, respectively. Xia Cao et al. revealed extraordinary progress with regard to capacity retention and columbic efficiency at elevated temperatures for LNMO/Li_4_Ti_5_O_12_ (LTO) cells containing LiTFSI/PYRTFSI electrolytes [89].

### 2.2. Pristine Ionic Liquids as Electrolytes

As we discussed earlier, the commercially available liquid electrolytes that consist of mixed organic solvents (linear and cyclic carbonates) and LiPF_6_ are highly flammable and thermally unstable at high temperatures [12,27,87,90]. To attain a maximum level of safety for LIBs, the progress of thermally stable electrolytes is the decisive one. For this, RTILs have been paid great consideration in the past due to their exceptional properties in terms of electrochemical and thermal stabilities [77,91]. Hence, in order to develop the solvent-free electrolytes, the RTILs, also known as “designer solvents”, have tunable properties, which is a valid alternative to replace carbonate solvents [21]. This section will thoroughly discuss the utilization of RTILs as “solvents” for dissolving lithium salts and acting as a pure electrolyte for LIB applications.

In the past decade, imidazolium and PYR-based ILs have become promising potential candidate for LIBs applications. These two IL families’ have a better ionic conductivity and low viscosity compared to other ILs and can allow the electrochemical intercalation as lithium into cathode materials, important industrial requirements for practical purposes. Some of the important niceties of these two families of ILs are discussed as follows: Archer et al. extensively studied silica nanoparticle-tethered Im_13_TFSI from the applied and fundamental perspectives. They revealed that the inclusion of nanoparticles in the electrolyte system hindered the crystallization of lithium salt (by means of the interactions between the TFSI counter-ion salt and the tethered cations of the IL surface), thereby enhancing lithium ion mobility, transference numbers, and the mechanical properties of the electrolytes [92,93].

Likewise, its analogous 1-ethyl-3-methylimidazolium bis(fluorosulfonyl)amide Im_13_FSI IL interaction with LiTFSI was elaborately and extensively analyzed through Raman spectroscopy by Fuji et al. [94,95]. They found that the lithium-ion solvation in FSI anion-based ILs is rather different from TFSI ion-based ILs, even though both anions have similar molecular structures and environments. Interestingly, the same anion interactions with PYR-based cations were experimentally and theoretically investigated by several groups [96,97,98].

Later, Deshpande et al. studied the flexibility and migration characteristics of lithium ions in PYR_13_TFSI ILs and characterized the electrolyte system using the molecular dynamics method [99]. These detected molecular dynamics and electrochemical (electrolyte ions mobility) results are in great agreement with the previous literatures using PYR as a cationic IL [100,101].

Based on the interactions between ILs and lithium salts as discussed above, several research groups have tried to enhance the other properties, such as the thermal, electrochemical, and cycling stabilities, for these kinds of liquid electrolytes [46,101,102,103,104,105,106]. The 1 M LiTFSI in PYR_14_TFSI electrolyte has showed better electrochemical and cycling properties with commercial liquid electrolytes [77] (1 M LiPF_6_ in EC/DEC).

Recently, IL-based electrolytes (LiTFSI-PYR_14_TFSI) have shown an optimized upper cut-off voltage in the range of 4.8−5.1 V and 3.2−3.6 V for metallic lithium and LTO-based dual-ion cells, respectively [107,108,109]. A similar type of enhancement has been observed for trimethylhexylammonium bis(trifluoro-methane sulfonyl)imide (TMHA-TFSI), glyme, and oligomeric ILs [2,109,110,111,112,113,114]. In contrast, Ferrari et al. prepared electrochemically stable and benign electrolytes for LIBs and Li-O_2_ batteries using 3-(2-(2-methoxy ethoxy)ethyl)-1-methylimidazolium TFSI (ImI_1,10201_TFSI) and 1-(2-methoxyethyl)-3-methylimidazolium TFSI (ImI_1,2O1_TFSI). But, the electrochemical stability windows were in a narrow range (<4 V), and no developments were observed in the cycling behaviors [31]. Table 2 presents the electrochemical and cycling properties of IL-based electrolytes.

### 2.3. Ionic Liquid-Based Gel Polymer Electrolytes

Commercial LIBs are comprised of LiPF_6_ in a mixture of carbonate solvents as the electrolyte [57,115,116]. When the cell operates, the anion present in lithium salt undergoes an equilibrium reaction and forms a Lewis acid PF_5_. This Lewis acid, in turn, reacts with the organic solvents EC/PC. The P–F bonds thus formed easily undergo hydrolysis, even if trace quantity of moisture is in the electrolyte medium, which, in turn, affects the performance of LIBs, hence making these solvents highly unsuitable [77,117,118,119]. Hence, in order to overcome the abovementioned issues, better alternatives of hydrophobic IL electrolytes are found to be one of the most promising electrolytes for LIBs, providing the stability for lithium electrode from air or moisture due to its hydrophobic-nature.

Apart from their chemical and electrochemical stabilities, some ILs are played as a wetness barrier, which is due to their trifling vapor pressure. Hence, the scientific community has been focused on decorating the concept of gel polymer electrolytes in electrolyte chemistry to incorporate IL moieties into polymer host structures, including poly(vinylidene fluoride) (PVdF) [120], poly (ethylene oxide) (PEO) [121]_,_ poly(vinylidene fluoride) hexafluoropropylene (PVdF-co-HFP) [51], poly(acrylonitrile) (PAN) [122], poly (methyl methacrylate) (PMMA) [123,124], polystyrene-block-poly methyl methacrylate-block-polystyrene (PS-b-PMMA-b-PS) [125], etc.

Further, gel polymer electrolytes (GPEs) satisfy numerous requirements, like high ionic conductivity at ambient temperature, compatibility with the electrodes, a broad electrochemical window, no leakage, better thermal and appropriate mechanical stabilities, etc., for their practical use in LIB cell. Furthermore, they possess two main advantages: firstly ‘‘solid-like’’ soft matter electrolytes with physicochemical properties superior to IL-based electrolytes without polymer host; and, secondly, lithium cell assembly also involves an only solid material, which is achieved by using the polymer as one of the medium.

**Figure 4:**
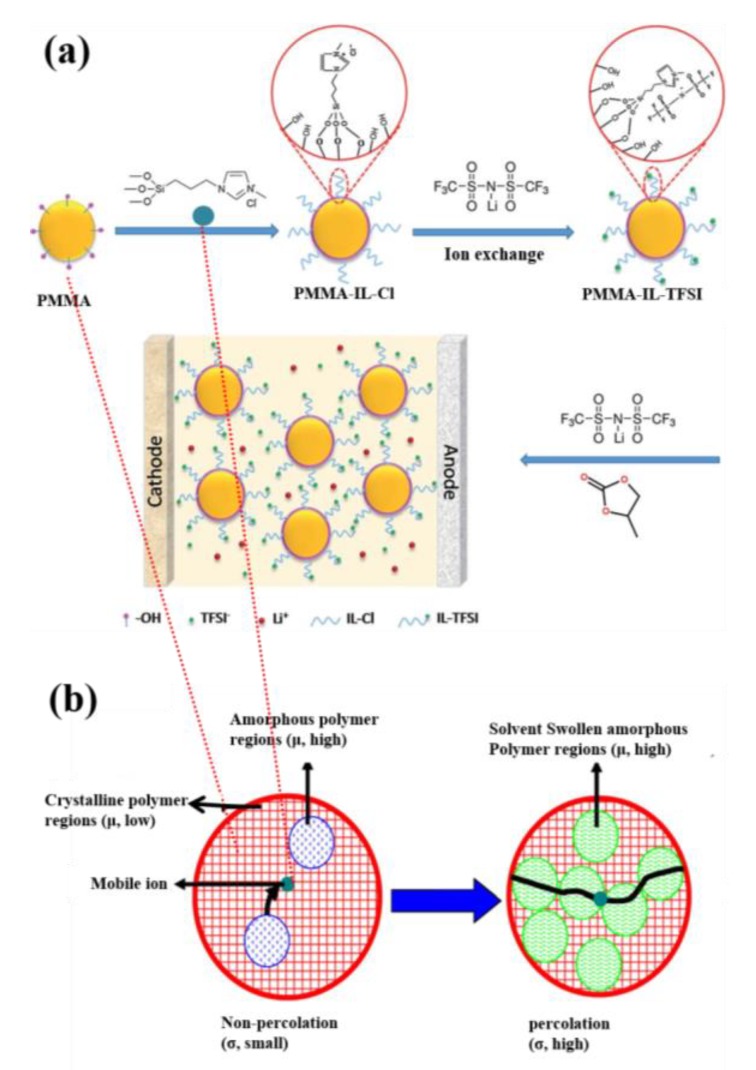
(**a**) Schematic illustration of gel polymer electrolyte (GPE) synthesis [123]. Copyright 2016 The Royal Society of Chemistry. (**b**) Percolation threshold mechanism for polymer electrolytes [126]. Copyright 2010 Elsevier.

The formation of a GPE using lithium ion conducting salt, IL, and PMMA polymer host is pictorially represented in Figure 4a [123]. The formation of PMMA-IL-TFSI-based electrolytes exactly follow the percolation threshold mechanism of polymers as reported earlier by Monalisa et al. [126]. It involved, at ambient temperatures, the concentration of amorphous regions well below the percolation threshold, which in turn lead to the lower value of ionic conductivity, as represented in the left-hand side of Figure 4b, and vice versa at the vicinity of melting temperature (T_m_), which is schematically represented on the right-hand side of Figure 4b.

Some of the recent literature strongly favors the utilization of ILs in GPEs to greatly enhance the conducting properties and cycling performance of the LIBs, which are summarized as follows. Patel et al. [127] investigated the room temperature IL gel polymer electrolyte LiTFSI-PYR_14_TFSI for application in LIBs using acrylonitrile monomer as the host. Interestingly, compared to pristine ILs, they observed better cycling performance and rate capability (current density 35–760 mAg^−1^) with LiFePO_4_ electronically wired with multi-walled carbon nanotubes (MWCNT) cathode, which indicates the polymer framework is capable of delivering excellent tracks for the rapid ions migration.

Likewise, a Pip_13_TFSI + LiTFSI/VC in a PVdF-co-HFP network exposed the maximum capacity of 340 mA h g^−1^ [128]. Rao et al. [124] prepared GPEs membrane based on electrospinning technique, which contained PAN/PMMA polymer host incorporated with an IL PYR_14_TFSI and delivered the supreme conductivity of 3.6 × 10^−3^ S cm^−1^, and it was unwavering for the high potential of more than 5 V vs. Li^+^/Li. The capacities of Li/GPE/LiFePO_4_ cells were found to be 139, 134, 120, and 101 mA h g^−1^ at the rate of 0.1, 0.2, 0.5, and 1 C, respectively, which is higher than previously reported PAN electrospun GPE membranes [129,130].

Recently, Liao et al. [130] constructed a half-cell Li/GPE 80 wt% PYR_13_TFSI/LiFePO_4_ that showed a discharge capacity of 90 mA h g^−1^ at 0.1 C, mainly owing to the intrinsically low conductivity of membrane at ambient temperature. In addition, they observed the improved cell performance by cycling at 50 °C, but it typically resulted in a lower columbic efficiency due to the increased sensitive nature of the electrolyte with anode.

ILs are purely responsible for the ionic transport/migration properties, as well as the amorphicity of the gel membranes. For instance, Shalu et al. prepared and examined PVdF-co-HFP-based ternary gel polymer electrolytes (TGPEs) using EMImBF_4_ and LiBF_4_, and their ionic conductivity, as well as the amorphicity, was enhanced with an increase in the concentration of Im_13_BF_4_ [40]. This may due to the decline of polymer crystallinity by the addition of Im_13_BF_4_ [131,132]. Similar kinds of behavior have been observed for other TGPEs containing ILs, such as Pip_13_TFSI [12,128]*,* PYR_1,4_TFSI [3,23,35], Pip_13_BETI [133,134], and Im_13_TFSI [134].

Recently, many research groups, including our group, have investigated the mechanism behind the transport properties of TGPEs using different types of ILs, which include Im_13_NfO [45], Im_14_NfO [44], Im_13_DFOB [135], Im_13_SCN [46] complexes with PVdF-co-HFP. In particular, Im_13_DFOB IL’s addition in PVdF-co-HFP significantly enhanced the room temperature conductivity (10^−5^ S cm^−1^), along with the stability window of 4.25 V. The transference number and cycling performance (148.4 mA h g^−1^) of the IL-GPEs increased drastically after EMImDFOB addition [135]. Pitwala et al. recently examined the consequence of various pyrrolidinium-based ILs, such as PYR_14_, PYR_24_, and TFSI, on the conducting and ion interaction properties of PVdF-co-HFP-based GPEs [34].

**Figure 5:**
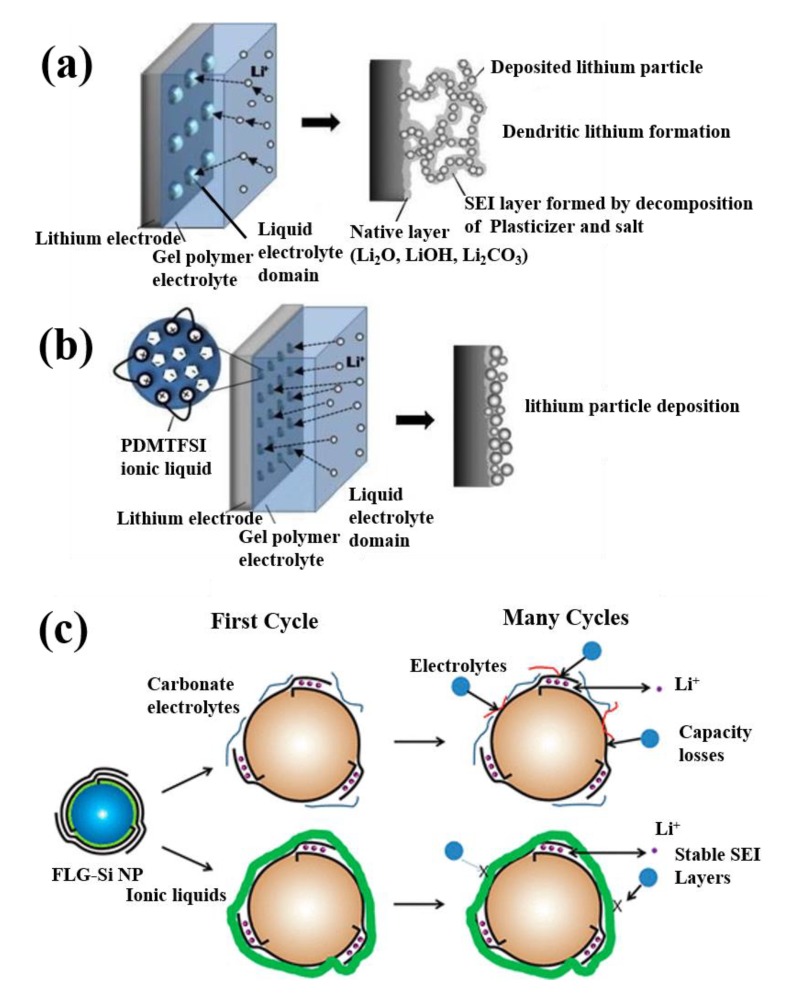
(**a**,**b**) Schematic illustration for the step by step functions of PDMITFSI IL on lithium deposition [137]. Copyright 2011 Elsevier. (**c**) Schematic illustration for the function of pyrrolidinium bis(fluorosulfonyl imide) on lithium deposition [138]. Copyright 2017 American Chemical Society.

The GPEs have been further extended by way of incorporating Al_2_O_3_ into the PMMA host, which are called quaternary polymer electrolytes (QPEs), drastically enhancing the thermal and cycling stabilities of the GPEs [91] by the way of crosslinking with the polymer host, which, in turn, to reduce the lithium ion (Li^+^) transport distance, thereby creates more lithium-ion conducting pathways. A similar type of quaternary polymer electrolyte (QPE) investigated by Li et al. using 1g_13_TFSI IL, LiTFSI, SiO_2_, and PMMA precursors. These prepared QPEs possessed thermal solidity (300 °C), robust electrochemical property with broad stability window (4.0 V), rapid ion migration property, interfacial stability, and excellent charge/discharge behavior due to the addition of nanosilica and 1g_13_TFSI [136]. Moreover, the anodic stability of the QPEs was increased enormously with the incorporation of dimeric ILs in the polymer host matrix [137], and it might be attributed to the formation of passive layers over the anode surface, which creates the massive growth of cells. Dissimilarly, the massive growth was not formed for a dense structure with uniform deposition of lithium. Hence, for anodic stability enhancement, it is important to minimize the formation of passive layers over anode, which is schematically illustrated in Figure 5a [137]. From Figure 5a, it is observed that it consists of two important phase regions (a) polymer-enriched medium and (b) liquid enriched medium. Figure 5a represents the carbonate solvent-based gel electrolytes which enhance the lithium ion concentration and deposit over the surface of the metal anode in a non-uniform manner to form a porous structure. The introduction of IL PDMITFSI in gel matrix occupies the interface of polymer enriched medium and the liquid electrolyte-enriched medium as shown in Figure 5b which in turn reduced the domains size of dispersed liquid electrolyte and increases the number of electrolyte-rich phase. This behavior is likely to boost the Li^+^ concentration in liquid electrolyte and thus lithium deposited uniformly in a particle-like a shape structure over the electrode surface and also regulate the dendrite free anode at the interface. A similar type of behaviors was observed by Park et al. [138] for PYR_1,4_TFSI IL electrolyte and few-layer graphene coated silicon anode (FLG-Si) electrode, as shown in Figure 5c. Due to the introduction of IL electrolyte, the passive layer formation on the anode surface is controlled and enhanced the capacity of the system drastically with a maximum of 4000 mAh g^−1^.

### 2.4. Ionic Liquid-Incorporated Solid Polymer Electrolytes

The bid of ILs to LIBs are not limited only for liquid and gel polymer electrolytes, and recently it has been widely constructed using solid polymer electrolyte. Unfortunately, the performance of the LIBs is restricted by the poor room temperature conductivity of solid polymer electrolytes, due to the crystallinity of polymers, which resulted in the decrement of migration rate of lithium ion species to the host matrix. This observation was evidenced by various results reported earlier [41,57,139,140,141]. The PEO-built solid electrolytes displayed an appropriate conductivity value (10^−4^ S cm^−1^) for commercial LIB usage only after their crystalline temperature (*T*_m_); in this case, the amorphous domains are dominant, which permits lithium ions to perceive a greater number of lithium charge carriers [41,120,142,143,144]. Thus, many attempts have been made, aiming to reduce the crystallinity, as well as to improve the robustness and conductivity in solid polymer electrolytes, which include blended matrices, branched hosts, bulky anionic lithium salts, and the incorporation of additives, such as fillers and plasticizers [145,146,147,148]. One such widely used facile method is to incorporate ILs into the polymer electrolytes, and it has been efficaciously demonstrated in recent years [22,43,85,102].

Recently, the incorporation of RTILs in polymer matrices has attracted considerable attention for their use as solid polymer electrolytes in rechargeable LIBs [24,37]. For instance, the PEO-based solid polymer electrolytes’ (SPEs) properties are hindered by the ILs’ addition in many ways. The major mechanism behind this phenomenon is explained as follows: the bigger anionic salts have the tendency to weaken the cations interaction from the ether oxygen coordination. The anions in ILs speed up the ionic migration, while the bulky cations are responsible for ionic conduction by means of creating unrestricted volume nearby the polymer lattice. Furthermore, the physical properties of the ILs, such as dielectric constant and viscosity, play a key role in electrolyte chemistry, which are the deciding factors for changing the physicochemical and electrical properties of the electrolyte films. The lower viscosity of the ILs and the higher flexibility in the polymer strand chain are increasing the conductivity rate in LIBs. Conversely, the higher dielectric constant value of ILs enhances the dissociation of paired ions, thereby increasing the mobile labile ions, which in turn favors the conductivity enhancement. A typical example of a PEO-based SPE comprising of a low viscosity (65 *cP*) and high dielectric constant (15) IL, namely EMImTf, in a PEO-LiTf complex has shown an extraordinary thermal stability (380 °C) and better ionic conductivity (3.1 × 10^−4^ S cm^−1^) due to the incorporation of low viscous IL [143].

A new class of IL, PYR_14_TFSI in a PEO matrix, subsequently enhances the chemical and electrochemical properties of SPEs [55], which are capable of providing the theoretical capacity (170 mA h g^−1^) at 30 °C through excellent cyclability. In a similar fashion, ILs, like PYR_14_TFSI, Im_13_TFSI, DEME-TFSI and PP_13_-TFSI, Im_13_TFSI, Im_23_PF_6_, etc., have been extensively studied and characterized for SPEs in LIB applications using PEO as the host polymer matrix [20,30,79,116,120,149,150,151,152]. Apart from PEOs, SPEs containing other polymer hosts have also been extensively studied [98,139,153]. A new class of SPE membranes, comprising IL PYR_1201_TFSI, BEMA and PEGMA, were prepared by the UV-induced photo-polymerization process, which showed a high anodic breakdown voltage of 4.6 V greater than Li/Li^+^ [33]. On the other hand, the electrochemical properties of the SPEs were enhanced with increasing concentration of PYR_14_TFSI and silicon fiber in the polycarbonate matrices. The tremendous increment was observed in lithium transference number (0.36), mechanical robustness, and cycling stability of these poly-carbonate membranes, which paves the way towards utilization as one of the potential candidates in rechargeable LIBs [154]. Additionally, in recent times, chitosan-based SPEs with fifteen different types of ILs were studied and identified as promising separators for LIBs [155]. Recently, polymeric IL (PoIL)-based SPEs have received great attention because of their chemical affinity which results in a compatible combination of stable polymer electrolytes. Li et al. [39,156,157] have prepared a series of SPE membranes containing different anions of guanidium protic ionic liquid (PIL), and it has possessed better thermal stability, with a decomposition temperature of 353 °C, and achieved the conductivity of 1.35×10^−4^ S cm^−1^ at 303 K. Likewise, poly((4-vinyl benzyl)trimethylammonium bis(trifluoro-methanesulfonamide))-based PILs have also been investigated in the recent years [154,155].

### 2.5. Ionic Liquid-Based Ionogel Electrolytes

Immobilizing ILs within organic, inorganic, or hybrid porous matrices lead to a solid-state ion-conducting ionogel system. The crucial role of ILs in ionogel system is maintaining their liquid dynamics, which is accountable for the ion conduction and electrochemical behavior, whereas the porous matrices offer abundant channels to confine the ILs and responsible for mechanical and optical properties.

Recently, solid-state lithium ion batteries with a new class of ionogel electrolytes using non-aqueous sol-gel synthesis based on a tetrabutyl titanate (TBOT) in an alkyl imidazolium IL like Im_13_BF_4_ and Im_13_TFSI have explored. They have reported a maximum room temperature ionic conductivity of 10^−3^ S cm^−1^, which is almost the same order as that is offered by organic liquid electrolytes and ILs [11]. Furthermore, the ionogel electrolytes were stable for long-time use more than one year, without any phase separation, and attained the highest specific capacity of 162 mA h g^−1^ at 1 C rate [98].

**Figure 6:**
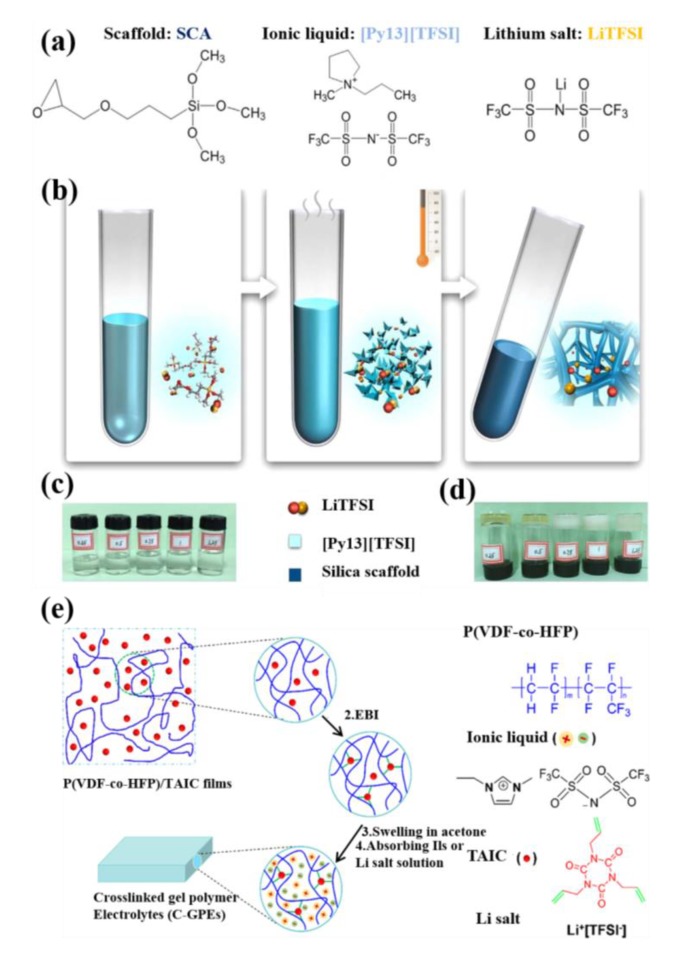
(**a**–**d**) Schematic diagram for non-aqueous sol-gel synthesis of ionogel electrolytes [158]. Copyright 2017 Elsevier (**e**) Schematic diagram for the synthesis of cross-linked GPEs [159]. Copyright 2017 American Chemical Society.

The non-aqueous sol-gel synthesis of ionogel electrolytes is schematically displayed in Figure 6a–d [158], and those prepared ionogel electrolytes delivered the capacity of 153.7 mAh g^−1^. The ILs with epoxy modified silanes formed organically modified silica-tethered solid state ionogels, which have superior ion-conducting and mechanical properties than to conventional solid polymer electrolytes. The synthesis process is exactly matched with the synthesis process of cross-linked gel polymer electrolytes using PVdF-co-HFP as polymer host reported earlier [159]. It is schematically represented in Figure 6e. It is important to notice that the difference between ionogel and GPEs is the host matrix; in the case of GPEs, the polymer acts as host, whereas, the case of ionogel electrolytes generally prefers siloxanes, epoxy, or silane scaffolds, which have been used. For instance, the recent report on 3-glycidyloxypropyltrimethoxy silane with PYR_13_TFSI/LiTFSI ionogel electrolyte has offered 1.91 × 10^−3^ S cm^−1^ at 303 K with a stability window of 4 V [158].

**Figure 7:**
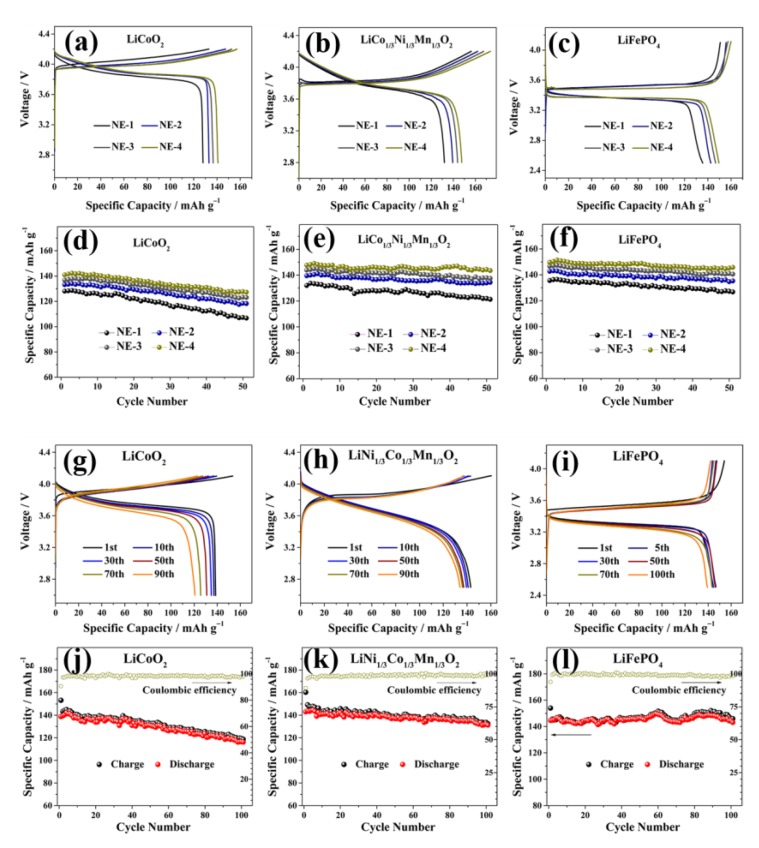
Electrochemical properties of ionogel electrolytes in half cells; initial CD profiles of LiCoO_2_/ionogel electrolytes/Li cells (**a**,**d**), LiCo_1/3_Ni_1/3_Mn_1/3_O_2_/ionogel electrolytes/Li cells (**b**,**e**), and LiFePO_4_/ionogel electrolytes/Li cells (**c**,**f**) functioned at 303 K and at 0.1 C. Electrochemical properties of ionogel electrolytes in ink jet printed ionogel electrolytes in half cells; CD performance of LiCoO_2_/ionogel electrolytes/Li cells (**g**,**j**), LiCo_1/3_Ni_1/3_Mn_1/3_O_2_/ionogel electrolytes/Li cells (**h**,**k**), and LiFePO_4_/ionogel electrolytes/Li cells (**i**,**l**) functioned at 303 K and at 0.1 C [160]. Copyright 2016 American Chemical Society.

Tan et al. employed ionogel electrolyte (BMImTFSI as the IL, LiTFSI as salt, HCOOH as the cross-linking agent and TEOS as the mesoporous matrices) for LIBs [160] in order to improve battery safety and cycle life. They assembled and fabricated the coin cells with two different approaches. The first approach involves the conventional method of battery assembly by the way of stacking the as prepared ionogel electrolyte on cathodes (they used three different electrodes, such as LiCoO_2_, LiNi_1.5_Mn_0.5_O_2_, and LiFePO_4_), followed by lithium metal anode to form a half cell. The second approach involves the inkjet printing of ionogel solvent precursors onto the as-prepared anodes and cathodes, stacked face to face in a coin cell to form a solid-state full cell. The resultant cell was then aged at 35 ºC for 24 h. In these two approaches, they analyzed the electrochemical and cycling performances both conventional (Figure 7a–f) and inkjet-printed electrolyte embedded cells (Figure 7g–l). The fuel cells prepared by ink jet printing show superior performance as compared to conventional half cells due to their better structure stability and interface compatibility between electrode and electrolyte. Hence, this approach of printing ionogel electrolytes onto the electrode surface allows fabrication of high-performance solid-state batteries with improved cycle performance and safety.

Earlier, a practical methodology employed for the enhancement of lithium mobility in boronic-ionogel electrolytes was using a highly soluble and crosslinkable boron allyl-oxide precursor as a chelating agent and the IL electrolyte solution PYR_14_TFSI/LiTFSI in the PEGDMA. The introduction of a boronic crosslinker in boronic ionogel electrolytes enhanced the lithium ion mobility more significantly than the ionogels fabricated without the boronic cross-linker, which leads to promising LIB performance, including both rate and cyclability, at elevated temperatures [161].

Polymer-supported ionogels have been hampered by two vital issues, which include the immiscibility between polymers and ILs and loss of the solid-state configuration of the polymers due to high loading of the ILs in polymers. These obstacles were overcome by preparing polymer-immobilized ionogel electrolytes with the mechanochemical activation process. A typical example would be an ionogel with a PEO scaffold and PYR_14_TFSI percolating phase investigated and observed at the maximum lithium ion conductivity of 7.28 × 10^−4^ S cm^−1^ without any conductivity decay over a long period of time [162]. A mixture of ILs, comprised of EMImTFSI and 3P(Im_14_TFSI) in PVdF-co-HFP electrospun films gave solid-like composite ionogel electrolytes and showed considerable tensile stability of 8.6 MPa and excellent thermal stability of 370 °C, which can fulfill the main prerequisite for safer energy storage devices, especially in LIBs [1].

## 3. Ionic Liquid-Based Electrolytes for Supercapacitor Applications

Ever increasing energy demand, depleting resources of fossil fuels, and increasing concern of climate change has motivated researchers to come out with greener and economically viable high-performance options for energy harvesting and storage devices [163,164,165,166,167,168,169,170,171]. Electrochemical energy and related technologies, which converts chemical energy into electrical energy without pollution, seems to be one of the effective way to address this problem. In recent times, SCs have demonstrated several promising characteristics, such as the capability of yielding high power, durable cycle life, and operational safety, and it is considered a next generation energy storage device.

Based on the charge storage mechanism, the two types of SC are: (1) the electrical double layer capacitor (EDLC), where the charge growth occurs at the interface between electrode and electrolyte; and (2) the pseudocapacitor, which uses quick and reversible redox reactions over the electrodes surface. To carry out these functions smoothly, most of the time, carbonaceous materials like carbon black, carbon nanotubes (CNTs), graphene, and activated carbon are employed as electrodes in EDLCs, while, in the case of pseudocapacitors, transition metal compounds (metal oxide) and/or conducting polymers are preferred to be used as electrode materials over the other materials [171,172,173]. EDLCs are having high rate charging-discharging capabilities in addition to high power density, superseding to batteries and fuel cells. In the EDLC, there is electrostatic adsorption of ionic species at the interface of electrode and solution. This leads to the formation of an electrochemical double layer, which ensures no redox reaction during the charging or discharging of these devices.

Among the various parameters, physical and chemical properties of electrolytes are one of the crucial factors to evaluate the performances of SCs. Efficient tuning of these properties is necessary to fabricate capable SCs. In general, aqueous electrolytes (e.g., H_2_SO_4_, KOH, Na_2_SO_4_) restricts the potential window, which is bounded by the cell voltage, energy, and electric power density. Although replacing these aqueous electrolytes by some organic and natural solvents ensures more stability over the wide range of potential, it certainly raised the issue of operational safety. Organic solvents are flammable, volatile, and sometimes even toxic, which can caused severe health issues. Consequently, the discovery of a more appropriate electrolyte is of massive importance to optimize the efficiency of SCs [4,174]. ILs, which have distinctive properties, like excellent thermal and chemical stabilities, wide electrochemical voltage window, reliable ionic conductivity, non-volatility, and nontoxicity, have been employed to overcome most of the problems associated with usage of liquid and organic electrolytes in SCs [17,175]. In the following sections, special focus is paid to the advances in IL-based electrolytes for applications in SCs. In addition, a detailed investigation is performed on the enhancement of electrochemical properties using IL electrolytes and polymer hybrid electrolytes, which are compared to other organic/aqueous electrolytes with energy storage devices in mind.

### 3.1. Pristine Ionic Liquids as Electrolytes

To realize the dream of greener and economically viable high-performance energy storage devices, a lot of efforts have been taken in designing and developing a new class of materials and technologies [4,176,177]. ILs have tunable physical and chemical properties; henceforth, it has a significant impact on the several of energy harvesting and storage-related technologies. As discussed in the previous section of this review, ILs are molten organic salts with melting points underneath 100 °C. It is observed that most of these IL salts have strong electrostatic forces between their molecular ions. As a result, most of them have low volatility/flammability and high chemical and electrochemical stabilities. These properties, along with high intrinsic ionic conductivity, are highly desirable as solvents and electrolytes for SCs. Earlier results suggested that the capacitance retention and operational safety at high temperatures is much better for SCs with ILs as electrolytes than those using non-aqueous electrolytes [11,25,75,178,179,180,181,182,183,184,185,186,187,188]. It is also observed that SCs fabricated using ILs can be operated at high cell voltage, which helps the improvement of energy density of SCs to the extent of secondary batteries [180,181,182,183,184,185,186,187,188,189,190,191,192,193].

ILs can be arranged into two groups: protic ILs (PILs) and aprotic ILs (AILs). Significantly, PILs are comprised of equimolar Bronsted acids and Bronsted bases. The proton exchange, from acid to base, makes proton donor and acceptor areas, which, in turn, can prompt the arrangement of H-bonds. PILs are simpler to blend, less expensive, less viscous, and have higher conductivities than AILs [65].

**Figure 8:**
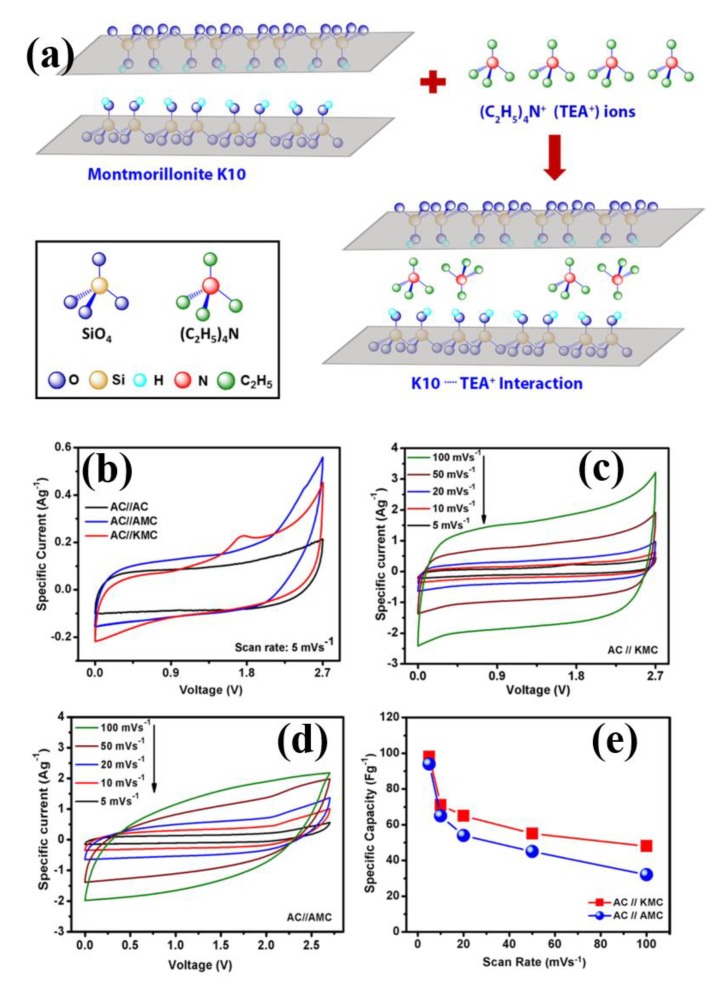
(**a**) Schematic mechanism of K10/MWCNT/MnO_2_ composite (KMC) with IL, (**b**) CVs of AC//KMC, AC//AMC and AC//AC SC cells at 5 mV s^−1^ scan rate, (**c**,**d**) CV plots of asymmetric cell with cell configuration AC//KMC and AC//AMC, (**e**) *C_sp_* vs. ν for AC//AMC and AC//KMC cells [193]. Copyright 2016 Elsevier.

Timperman et al. [65] prepared phosphonium PIL [Bu_3_HP][BF_4_] and investigated as the electrolyte for carbon-SCs. Kuring et al. [189] investigated a series of different types of ILs, such as Im_13_ cation and BF_4_, TCB, TFSI, FSI, and SCN anions, as electrolytes in asymmetric SC cells. They observed the IL electrolytes have a wide electrochemical stability window and better capacitance performance than organic electrolytes. Denshchikov et al. [190] fabricated an SC device using nanostructured carbon electrodes and solvent-free IL 1Me_3_BuImBF_4_ as electrolytes with the extreme specific capacitance of 111 F/g at ambient temperature. Shaikh et al. [191] fabricated inexpensive and nontoxic Ru-doped CuO film as an electrode and Bronsted acid, 1-butyl-3-methylimidazolim bisulfite (Im_14_HSO_4_) as an electrolyte for SCs. In addition, they achieved the capacitance of 406 F.g^−1^ at low scan rate for 15 volume percentage Ru-doped CuO film. Anouti et al. [192] investigated the two PILs, such as pyrrolidinium methanesulfonate, PYRMeSO_3_, and diisoprpylethylammonium methanesulfonate (DIPEAMeSO_3_), in the water mixture as the electrolytes with activated carbon as electrodes and observed the specific capacitance of 97 F g^−1^ with the specific power of 13.9 kW kg^−1^ at a high current density of 15 A g^−1^. In addition, Maiti et al. [193] used IL Et_4_NBF_4_ electrolyte soaked separator between the montmorillonite K10 clay-MWCNT-MnO_2_ composite electrodes and achieved 171 W h kg^−1^ and 96.4 kW kg^−1^ as the energy and power densities, respectively, which is represented in Figure 8a–e. The acid-base interactions of K10 via its surface OH groups with the IL electrolyte (C_2_H_5_)_4_N^+^ or TEA^+^ could be the possible reason for observed high performance of EDLC.

In general, as acetonitrile is a preferred solvent for electrolytes which has low viscosity and high conductivity with the mixture of salts: however, it possesses the issue of operational safety owing to their high volatility and flammability. Abdallah et al. [194] successfully replaced aprotic tetraethylammonium tetrafluoroborate (Et_4_NBF_4_) ILs-acetonitrile in SC applications and proved their less volatile and flammable nature, which may be due to the presence of solvates. Reduced graphene oxide (RGO)-based SCs were fabricated by Chen et al. [195] using IL electrolytes of Im_14_PF_6_ and Im_14_BF_4_. They were observed the specific capacitance of 74 F g^−1^ for the fabricated SC with RGO in Im_14_BF_4_ at 10 mV s^−1^, which is much better than the specific capacitance of 45 F g^−1^ for SC with RGO in Im_14_PF_6_.

Demarconnay et al. [15] demonstrated the SCs using PYRNO_3_-based electrolytes and carbon electrodes with the specific capacitance of 121 and 208 F g^−1^ for their pH value of 7 and 11, respectively. In this case, the triethylammonium bis(trifluoromethylsufonyl)imide–NEt_3_H TFSI–PIL was employed to broaden the electrochemical window. In addition, Brandt et al. [192] investigated the role of water content in protic IL electrolyte on its performance for SC applications. They found that devices have the excellent stability, even at different temperature range, with the operative voltage of 2.4 V. Furthermore, they observed the pseudocapacitive behavior of an AC (Activated Carbon) electrode in protic IL electrolyte due to the water content. Trigueiro et al. [7] prepared the SC using an RGO as an electrode and PYR_13_TFSI as the electrolyte, which showed the specific capacitance of 71.5 F g^−1^ at 10 mV s^−1^.

The operating potential window, which is generally governed by electrolyte, is one of the important parameter that can affect the specific energy of EDLCs. ILs have been paid attention to as electrolytes for EDLCs because they tend to have comparatively broad potential windows compared to organic electrolytes. The maximum specific energies were attained with IL electrolytes that have reasonable electrochemical stability, low viscosity, small ionic volumes, and therefore high ionic conductivity. Yang et al. [196] studied graphene nanosheets to enhance SC behavior in a butylmethylpyrrolidinium–dicyanamide (PYR_14_DCA) IL electrolyte. Shao et al. [197] utilized IL (EMIMBF_4_) electrolyte and CNT spaced graphene aerogels electrodes for SCs, which offered the highest capacitance of 183.3 F g^−1^ at 0.5 A g^−1^ and the energy density of 80 W h kg^−1^. Fuertes et al. [198] fabricated an SC using EMImTFSI as the electrolyte and porous carbon electrode with a specific capacitance of 160 F g^−1^ at 1 A g^−1^.

Kim et al. [187] successfully achieved an ultrahigh energy densities in assembled SCs due to the excellent pore structure of CNF-based electrodes in IL electrolyte systems. Jha et al. [199] reported a specific capacitance of 222 F g^−1^ for SCs using an IL Im_14_BF_4_ electrolyte. Further, the similar type of BF_4_ anion-based IL electrolyte provides better performance with other carbonaceous materials as reported elsewhere [32,197,199,200,201]. Iamprasertkun et al. [6] demonstrated a 1-butyl-1-methylpyrrolidinium dicyanamide, [BMP][DCA] as potential electrolyte for N-rGO aerogel electrodes-based SCs, which delivered the specific capacitance of 764.53 F g−1 at 1 A g^−1^. The electrochemical and cycling properties of pristine IL-based electrolytes are tabulated in Table 3 [201,202,203,204,205,206,207,208,209,210,211,212,213,214,215,216,217,218,219,220,221,222,223,224,225,226,227,228,229,230].

**Figure 9:**
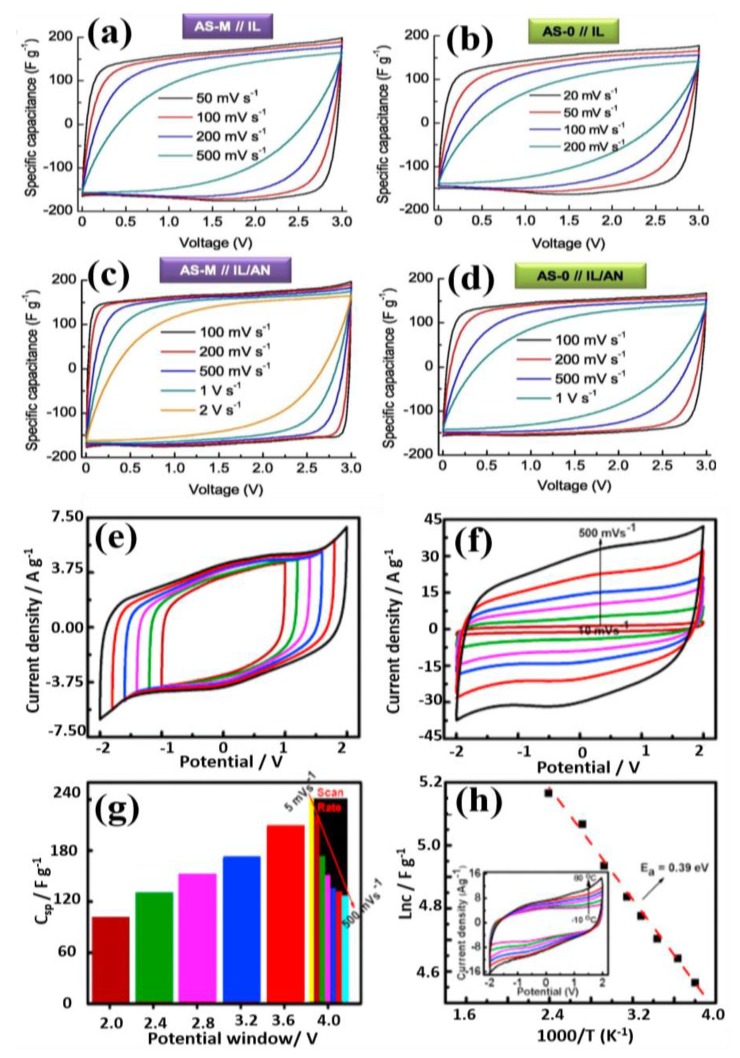
CV curves of pure EMImTFSI (**a**,**b**) and EMImTFSI/acrylonitrile (AN) (**c**,**d**) using sawdust carbon symmetrical electrodes at room temperature; CV curves (**e**) different potential window and (**f**) different scan rate 10–500 mV s^−1^, (**g**) capacitance variations with potential window, (**h**) Arrhenius plot of specific capacitance (Inset: temperature dependent CV curve) for *f*-graphene in BMImBF_4_ electrolyte and gel electrolytes [198,234]. Copyright 2017 Elsevier.

Using Im_14_NTf_2_ electrolyte, Bettini et al. [231] assembled the SC with cluster-assembled nanostructured carbon electrodes, which showed the maximum capacitance of 75 F g^−1^. Deng et al. [163] developed the MnO_2_ nanowires/Ni foam as an electrode and the new type of lithium-ion quasi-IL (LiClO_4_/2-oxazolidinone (OZO) salts) electrolyte for pseudocapacitors, which operated in the broad potential range (2.5 V) with 304 W h kg^−1^ energy density. Fu et al. [222] got the capacitance of 132 F g^−1^ with the specific energy and power density of 143.7 W h kg^−1^ and 2.8 kW kg^−1^, respectively, for IL Im_14_PF_6_ in acetonitrile electrolyte. Fuertes et al. [198] studied the effect of Im_14_TFSI at different temperatures using sawdust carbon electrodes, and it showed apparently very good capacitance behavior. Its corresponding CV curves are displayed in Figure 9a–d. Tooming et al. [228] examined the effect of EMImI (5 wt%) on EMImBF_4_ as the electrolyte on the electrochemical performances in EDLC. They observed that specific conductivity decreased to 12.8 mS cm^−1^ from 13.6 mS cm^−1^ due to the incorporation of 5 wt% EMImI into the EMImBF_4_ with the improved melting point of 19.5 °C. Zhang et al. [227] reported the specific capacitance of 523.3 F g^−1^ at 3 A g^−1^ with the specific power of 20.4 kW kg^−1^ at a specific energy of 8.5 W h kg^−1^ for Im_14_PF_6_/N, N-DMF electrolyte and MnO_2_ electrodes. Sathish et al. [225] attained the capacitance of 104 F g^−1^ using N-doped GN and Im_13_TFSA as the electrode and electrolyte, respectively.

Xie et al. [232] investigated redox-active IL as electrolyte for SC applications where the IL was utilized with ferrocene as an electroactive material to enhance the energy density, which led to high concentrations of redox moieties in the electrolyte (2.5 M) and a wide working potential, around 2.5 V. Lin et al. [233] achieved a highest specific capacitance of 130 F g^−1^ (63 mA h g^−1^) at −20 °C, 100 F g^−1^ (49 mA h g^−1^) at −30 °C and 175 F g^−1^ (85 mA h g^−1^) at 80 °C for fabricated SC using an eutectic mixture of Pip_23_TFSI and PYR_24_TFSI IL electrolyte with graphene film. Tsai et al. [188] employed a eutectic mixture of ILs, such as Pip_13_FSI and PYR_14_FSI, as an electrolyte with exfoliated graphite oxide electrodes, which reached 180 F g^−1^ in the voltage window of 3.5 V at 20 mV s^−1^. Furthermore, various novel ILs, which, comprising different anions, such as BF_4_, triflate, and TFSI, as well as cations, such as ammonium, Im, Pip, PY, and PYR, are used as electrolytes for SCs [8,32,200]. ILs presents a distinctive set of behaviors, which made them promising material for electrochemical SC devices. The development of ILs in SC applications is promising but also presents several challenges.

### 3.2. Ionic Liquid Based Polymer Electrolytes

The liquid electrolytes, including ILs, are capable of the fabrication of SC, but they have limitations due to their requirement of encapsulation, which limits their use in flexible and printed electronics [47]. The IL-based (gel) polymer electrolytes (GPEs) are suitable alternatives to address this issue in IL-based electrolytes for flexible energy storage devices [205]. The incorporation of ILs and their associated functional groups (i.e., cations, like pyrrolidinium, pyridinium, and imidazolium; and anions, like triflates, hexafluorophosphate, and tetrafluoroborate) with well-designed polymers are creating a new avenue of materials, which has fascinating properties and suitable for a huge range of applications. By appearing in their chemical structure, IL incorporated polymers are polyelectrolytes with restating polymers group stance as the electrolyte (cation/anion). In GPE, polymeric network ensures mechanical reliability, which makes them more mechanically stable. However, GPE systems which comprised of liquid electrolyte with organic solvent are showed inferior electrochemical stability with narrow potential window and low thermal stability due to the flammable nature of organic solvents [205].

Ujjain et al. [234] assembled a SC with a high specific capacitance of 242 F g^−1^ at 5 mV s^−1^ and stability up to 12,000 cycles using modified RGO electrodes with an IL of Im_14_BF_4_ and GPE of Im_13_BF_4_/[PVdF-co-HFP] as shown in Figure 9e–h. Tamilarasan et al. [235] fabricated an SC using high strain stretchable IL-integrated Im_14_TFSI/PMMA electrolyte and graphene electrode. Similarly, Lee et al. [236] fabricated as str*etc*hable SC using PVdF-co-HFP/IL electrolyte and PDMS/CNT electrodes. This SC cell possessed superior capacitance retention of 96.6% over 3000 cycles. The PVdF-co-HFP/IL GPE improves the operating potential window and the energy density. In addition, Tamilarasan et al. [122] observed 98 F g^−1^ of specific capacitance at 10 A g^−1^ for SC using IL-integrated polymer electrolytes (PAN/Im_1,4_TFSI) with the specific power and energy density of 82 kW kg^−1^ and 32.3 W h kg^−1^, respectively.

Pandey et al. [5] synthesized GPEs using the hydrophobic IL of (1-ethyl-3-methylimidazolium tris(pentafluoroethyl) trifluorophosphate) (Im_13_FAP) incorporated in PVdF-co-HFP, and they showed the broad electrochemical window of 4.4 V using MWCNT electrodes with the 2 × 10^−3^ S cm^−1^ electronic conductivity at 298 K, 76 F g^−1^ specific capacitance at 1.0 mA cm^−2^, 17.2 W h kg^−1^ energy density, and 18.9 kW kg^−1^ power density. The highest ionic conductivity of 7.31 mS cm^−1^ was attained at 393 K by Liew et al. [237] due to incorporation of 50 wt% of PVA/CH_3_COONH_4_/Im_14_Cl-based polymer electrolytes with considerable SC parameters (specific capacitance—31.28 F g^−1^; energy density—2.39 Wh kg^−1^; power density—19.79 W kg^−1^). Pandey et al. [205,207] fabricated an SC based on PEDOT-coated carbon fiber paper as the electrode and Im_14_BF_4_ IL-based PVdF-HFP gel polymer electrolytes, which provided the maximum capacitance value of 154.5 F g^−1^ at 1 mA cm^−2^.

Recently, flexible SCs were constructed using CNTs and IL-based GPEs, and their capacitance value was 135 F g^−1^ at 2 A g^−1^ [204]. Pandey et al. [5] studied IL Im_13_FAP-incorporated GPEs and MWCNT electrodes for SC applications.

In addition, polymer electrolytes, such as PYR_1,4_TFSI (poly (diallyldimethylammonium) bis(trifluoromethanesulfonyl) imide (pDADMATFSI) and N-butyl-N-methyl pyrrolidinium bis(trifluoromethylsulfonyl) imide in the ratio of 40:60, w/w), succinonitrile (SN) and Im_14_BF_4_-incorporated PVdF-co-HFP gel polymer electrolytes, and 1-ethyl-3-methylimidazolium tetracyanoborate (Im_13_TCB)-incorporated PVdF-co-HFP polymer GPEs, have been successfully used in SC applications [14,47,238,239,240,241,242,243,244,245,246,247]. Liew et al. [14] prepared IL 1-butyl-3-methylimidazolium trifluoromethane sulfonate (Im_14_Tf)-based PVA/ammonium acetate polymer electrolytes by the solution casting method. Furthermore, the prepared electrolytes were utilized in carbon-EDLC SCs and obtained the highest power density of 18.37 kW kg^−1^. Table 4 summarizes the cycling and electrochemical properties of different IL incorporated GPEs for SCs application.

Pandey et al. [143,246] assembled a symmetric EDLC with activated charcoal and magnesium-incorporated Im_13_Tf/PVdF-co-HFP GPE, and their capacitance, energy density and power density value were found to be 136 F g^−1^, 18.8W h kg^−1^, and 6.67 kW kg^−1^, respectively. The overall observation is that ILs have a strong ability to make efficient energy storage devices as SCs. Nevertheless, more remains to be done, with a clear focus on the benefits of SC applications.

## 4. Summary and Outlook

In this review, we outlined the recent developments of liquid, gel, and solid IL-based electrolytes for their applications in LIBs and SCs. In particular, discussions were focused to highlight the excellent electrochemical and physicochemical properties of some organically modified electrolytes with ILs for their applications in energy storage systems. Today, the significance of EES materials is increasing due to their huge requirements. Hence, many new class of ILs and polymeric ILs have been developed for LIBs and SCs that provide high-performance devices with excellent specific capacity and capacity retention properties.

Some of merits associated with the use of ILs for fabricating EES devices are as follows: (a) the characteristic properties of IL, includes thermal stability, ionic conductivity, and negligible vapor pressure certainly matches with the industrial requirements; (b) the low volatility and better electrochemical stability of ILs makes them ideal choice as electrolytes for LIBs and SCs; (c) improved energy density and capacity maintenance, hence making it is the key material for large-scale energy storage systems; and (d) the excellent unique feature of ILs, observed from most of the literature, is its capability to operate at elevated temperatures, which makes them the promising candidates for applications in portable electronic devices and hybrid electric vehicles. Along with the polymer host, ILs can be used to formulate solid and gel electrolytes; hence, it would be a capable material for many applications, like flexible electronics, batteries, wearable, fuel cells, bendable SCs, and DSSCs.

## Figures and Tables

**Table 1:** Electrochemical properties of ILs and carbonate-solvents based liquid electrolytes.

ILs	Carbonate Solvents	σ (mS cm^−1^)	Electrochemical Stability Window (ESW) (V)	*C* (mA h g^−1^)	Cathode	Ref
PYR_14_TFSI	EC, DEC & VC	2.4	>4.8	148	LiNi_1/3_Co_1/3_Mn_1/3_O_2_	[57]
PYRNO_3_	PC	30	1.5	134	Carbon coated LiFePO_4_	[64]
AMImTFSI & Im_13_TFSI	PC	7.8	5.8	144.2	LiFePO_4_	[83]
PYR_14_TFSI	EC: DMC	--	6.5	180–200	LiFePO_4_	[36]
PYR_14_TFSI	PC	5.1	4.2	145	LiFePO_4_	[52]
DMMATFSI	VC	>1	>4.5	142	LiNi_1/3_Co_1/3_Mn_1/3_O_2_	[85]
Pip_13_TFSI	PC	10	4.35	2230	3D-porous silicon	[88]
PYR_13_TFSI	PC, VC	>5	5	160	LiFePO_4_	[48]
MMMPYRTFSIPYR_14_TFSI	EC, DMC	--	5.2–5.5	118	LiNi_0.5_Mn_1.5_O_4_	[89]
Pip_13_TFSI	DEC	10^2^	5.4	146	LiCoO_2_	[53]
PYR_14_TFSI	EC	10^−3^	4.8	164	C/LiFePO_4_	[56]
PYR_14_TFSI	VC, FEC, EC	--	5.3	148	LiNi_1/3_Co_1/3_Mn_1/3_O_2_	[57]
MEMBu_3_PTFSI; Pip_13_TFSI	PC	--	5.1–5.5	3450	gas deposited Silicon	[90]
PYR_14_TFSI	EC	10	1.4	353	graphite	[84]
Pip_13_TFSI	DME	---	2.4	1670	Ketjenblack (KB) carbon	[69]
Im_13_TFSI	EC, DC	>10^2^	4.5–5	---	---	[73]
Im_13_TFSI	EC	7.34	--	--	---	[79]
PYR_14_TFSI	PC	11	2	140	LiFePO_4_	[81]
PYR_13_TFSI	EC	--	>5	148	LiFePO_4_	[54]
PYR_13_TFSI	EC, DEC	10	---	102	LiFePO_4_ & Li_4_Ti_5_O_12_	[86]

**Table 2:** Electrochemical performances of pristine IL incorporated electrolytes.

ILs	σ (mS cm^−1^)	ESW (V)	*C* (mA h g^−1^)	Cathode	Ref
Im_13_TFSI- SiO_2_	10^4^	>4.25	--	--	[92]
Pip_13_TFSI-SiO_2_	10^3^	--	154	Li_4_Ti_5_O_12_	[93]
PYR_13_FSI; P_111i4_FSI	1.50	--	130	LiNi_1/3_Mn_1/3_Co_1/3_O_2_	[97]
Im_13_TFSI	3.8 × 10^3^	2	150	LiFePO_4_	[98]
PYR_14_FSI and	3.3 × 10^3^	4.0	135	LiFePO_4_	[100]
PYR_14_TFSI	10^3^	4.9	160	LiFePO_4_	[100]
TMHATFSI	4.5 × 10^5^	3.0	675	Hard carbon	[2]
QATFSI	--	1.6	2000	Si/LiCoO_2_	[38]
IMI_14_TFSI	4.38 × 10^3^	3.9	167	LiFePO_4_	[31]
IMI_1,2O1_TFSI	4.26 × 10^3^	4.4	--	LiFePO_4_	[31]
IMI_1,10201_TFSI	1.88 × 10^3^	3.6	--	LiFePO_4_	[31]
Pip_I3_FSI	3.85 × 10^3^	3–5	>360	graphite	[114]
PYR_14_TFSI	--	4.5–5	110	LiNi_0.5_Mn_1.5_O_4_	[77]
PYR_13_FSI	--	2.2	120	LiCoO_2_	[22]
PYR_14_TFSI	--	1.2	100	graphite	[107]

**Table 3:** Electrochemical and cycling properties of pristine IL-based electrolytes for applications in SCs.

Pristine Ionic Liquids as Electrolytes				
Ionic Liquids	Working Electrodes Used	C_sp_ (Fg^−1^)	ESW (V)	Ref
Im_14_BF_4_	AC	111	3.5	[190]
Im_14_PF_6_	Reduced graphene oxide (RGO)	158	1	[202]
Im_14_PF_6_ & Im_14_BF_4_	RGO	74 45	4 3	[195]
Et_4_NBF_4_ & TMPA-TFSI	Pt/Ag	85.9 90.9	5 5.5	[194]
DIPEAMeSO_3_; PYRMeSO_3_	AC	88 97	1.5 1.5	[203]
Im_13_TFSI	Carbon nanotubes (CNTs)	135	3	[204]
(1-ethyl-3-methylimidazolium Im_13_FAP	multi-walled carbon nanotubes (MWCNT)	127	4	[205]
PYRNO_3_	AOX Carbon (Resorcinol-formaldehyde organic xerogel)	208	1.2	[15]
Im_14_BF_4_	KOH treated Carbon-Xerogel	--	1.1	[206]
Im_13_PF_6_	MWCNT	76	4.4	[5]
Pip_13_FSI PYR_14_FSI	KOH-activated microwave exfoliated graphene oxide (α-MEGO)	180	3.5	[188]
Et_3_NHTFSI Me_3_NHTFSI-PC	AC	20.2 16.8	2.1 2.25	[192]
Im_14_BF_4_	PEDOT coated carbon	154.5	1.2	[207]
Et_3_NHSO_4_	Graphite	140	3	[208]
Im_14_FeCl_4_	g-C_3_N_4_ hybridized α-Fe_2_O_3_	265	1	[209]
MPPYRTFSI	protic ionic liquid (PIL) treated RGO	71.5	3	[7]
Im_34_PF_6_	Graphene	114	1.1	[210]
Im_13_BF_4_/Ammonium peroxydisulfate	PANI treated Carbon	565	0.8	[211]
PYRTFSI	AC	100	1.5	[212]
PYR_1,4_DCA	Graphene nanosheets (GNs)	330	3.3	[196]
PYR_1,4_DCA	Graphene nanosheets (GNs)	235	3.3	[213]
Im_13_BF_4_	Glucose derived AC	158	3	[214]
Im_13_BF_4_	CNT spaced Graphene aerogels	245.5	1	[197]
PYR_13_ BTA/LiClO_4_	3D-hierarchical ultrathin MnO_2_ nanoflakes@ SiNWs	51.46	2.2	[215]
PDADMATFSI/PYRTFSI	AC	100	3.5	[47]
P-Benzoquinone in PYRTFSI	AC	156	3	[8]
Et_4_NBF_4_	K10/MWCNT/MnO_2_	100	2.7	[193]
Im_13_BF_4_	Soybean root derived 3D-hierachical porous carbon	276	1	[216]
Im_14_Im	Carbon nanosheets	341	1	[217]
Im_13_BF_4_	Polypyrrole derived AC	300	4	[218]
Im_14_BF_4_	SWCNT & RGO	222	3.5	[199]
Im_13_BF_4_	Hexagonally ordered mesoporous carbon	203	3	[219]
Im_13_TFSI/PIL	PIL/RGO	187	3.5	[220]
Im_16_BF_4_	Polybenzoxaxine co-resol-based hierarchically porous carbon	247	1	[221]
Im_14_PF_6_	RGO	132	3	[222]
PYR_13_TFSI	Carbon Films	102	2.5	[223]
Im_13_TFSI	3D-organically modified carbon	146–178	3.5	[224]
Im_13_TFSI	N-doped GNs	104	3.6	[225]
Me_3_STFSI/PC	AC	25	3	[226]
Im_14_PF_6_/DMF	MnO_2_	523.3	3	[227]
Im_13_BF_4_	GO-CMK-5	144.4	3.5	[201]
Im_13_I+Im_13_BF_4_	Micro-mesoporous carbon	245	1	[228]
PYRTFSI+DIPEATFSI	AC	120	2	[229]
PYRTFSI	Polyfluoroalcohol activated AC	150–160	1	[230]

**Table 4:** Electrochemical and cycling behaviors of different IL incorporated GPEs for applications in SCs.

Polymer Gel Electrolytes	Electrodes Used	C_sp_ (Fg^−1^)	ESW (V)	References
PMMA/Im_14_TFSI	Graphene	83	3	[235]
PAN/Im_14_TFSI	Graphene	128	3	[122]
Chitosan/Im_13_BF_4_	Carbon fiber	130	2.5	[239]
PVA/Im_14_Cl/CH_3_COONH_4_	AC	30	3.7	[237]
PVdF-co-HFP/Im_13_TFSI	Graphene Nanosheets	96	3	[240]
Im_14_BF_4_, Im_13_BF_4_/PVdF-co-HFP	Graphene	242	4	[234]
PVA/Im_14_Br/NH_4_COOCH_3_	AC	21.89	1.1	[241]
PVdF-co-HFP/Im_14_DCA	MWCNT	2.2–3.5	3.5	[242,243]
PVA/CH_3_COONH_4_/Im_14_TFSI	CNT	31	3.3	[14]
PVdF-co-HFP/SN/Im_14_BF_4_	AC	176	2.5	[238]
PVdF-co-HFP/Im_13_BF_4_	GO	140	3.5	[244]
PVdF-co-HFP/Im_13_Tf/MgTf	AC	136	3.5	[245]
PVdF-co-HFP/Im_13_TCB	MWCNT	34.4	3.8	[246]
PVdF-co-HFP/Im_13_Tf/MgTf	MWCNT	32–41	2	[247]

## Abbreviations

	**Name**
AIL	Aprotic ionic liquid
AMImTFSI	1-allyl-3-methylimidazolium bis(trifluoromethanesulfonyl)imide
BMImBF_4_	1-Butyl-3-methylimidazolium tetrafluoroborate
PYR_14_TFSI	N-butyl-N-methyl-pyrrolidinium bis(trifluoromethanesulfonyl) imide
Im_14_NfO	1-butyl-3- methylimidazolium bis(nonafluorobutane sulfonate)
DMMATFSI	N,N-diethyl-N-methyl-N-(2-methoxyethyl)ammonium bis(trifluoromethylsulfonyl)imide
DME; DMF	dimethoxy ethane; dimethyl formamide
DEC	Diethyl carbonate
DMC	Dimethyl carbonate
DEMETFSI; DIPEATFSI	Diethylmethyl(2-methoxyethyl)ammonium bis(trifluoromethanesulfonyl) amide; Diisopropyl-ethyl-ammonium bis (trifluoromethanesulfonyl) amide
DSSCs	Dye Sensitized Solar Cells
Im_13_C(CN)_3_	1-ethyl-3-methylimidazolium tricyanomethanide
Im_13_Tf; LiTf; MgTf	1-ethyl-3-methylimidazolium bis(trifluoromethane sulfonate); Lithium bis(trifluoromethane sulfonate); Magnesium trifluoromethane sulphonate
Im_13_B(CN)_4_	1-ethyl-3-methylimidazolium tetracyanoborate
Im_13_SCN	1-ethyl-3-methylimidazolium thiocyanate
Im_13_BF_4_	1-ethyl-3-methylimidazolium tetrafluoroborate
Im_13_N(CN)_2_	1-ethyl-3-methylimidazolium dicyanoamide
Im_13_DFOB	1-ethyl-3-methylimidazolium difluorooxalato borate
Im_13_FAP	1-ethyl-3-methylimidazolium tris(pentafluoroethyl) trifluorophosphate
Im_13_NfO	1-ethyl-3-methylimidazolium bis(nonafluorobutane sulfonate)
EES	Electrochemical Energy storage
EMImFSI	1-ethyl-3-methylimidazolium bis(fluorosulfonyl)amide
EMImBF_4_	1-Ethyl-3-methylimidazolium Tetrafluoroborate
EDLCs	Electric double layer capacitors
EC	ethylene carbonate
FSI	Bis(fluorosulfonyl imide)
FEC	fluoroethylene carbonate
GPEs	Gel polymer electrolytes
IL	Ionic liquid
Im_2,3_PF_6_	1-ethyl-3-propylimidazolium hexafluorophosphate
GO-CMK-5	Negatively charged graphene oxide with positively charged mesoporous carbon CMK-5 platelets
ImI_1,201_TFSI	1-(2-methoxyethyl)-3-methylimidazolium bis(trifluoro-methane sulfonyl)imide
ImI_1,10201_TFSI	3-(2-(2-methoxyethoxy)ethyl)-1-methylimidazolium bis(trifluoro-methane sulfonyl)imide
Im_13_TFSI	1-methyl-3-propylimidazolium bis(trifluoromethanesulfone) imide
Im_14_TFSI	1-butyl-3-methylimidazolium bis(trifluoro-methane sulfonyl)imide
LIBs	Lithium-Ion batteries
LiNO_3_	Lithium nitrate
LiTFSI	Lithium bis((trifluoromethanesulfonyl imide)
LFP	Lithium iron phosphate
LiBOB	lithium bis(oxalato) borate
LTO	Lithium titanate
LiBF_4_	Lithium tetrafluoroborate
LiPF_6_	Lithium hexafluorophosphate
LiMNC	Lithium manganese nickel cobalt oxide
MMMPYRTFSI	methyl−methylcarboxymethyl pyrrolidinium bis- (trifluoromethane-sulfonyl)imide
MEMBu_3_PTFSI	1-methoxyethoxymethyl(tri-*n*-butyl)phosphonium bis(trifluoromethanesulfonyl)amide
MWCNT	Multi-walled carbon nanotubes
MEMBu_3_PTFSI	1-butyl-3-methylphosphonium bis((trifluoromethanesulfonyl imide)
MPPYRTFSI	N-methyl-N-propylpyrrolidinium bis(trifluoromethanesulfonyl) imide
PIL	protic ionic liquid
PYR_24_TFSI	*N*-butyl-*N*-ethylpyrrolidinium bis(trifluoromethanesulfonyl) amide
Pip	1-methyl-1-propylpiperidinium bis(trifluoromethanesulfonyl)-imide
PYR_14_TFSI	1-butyl-3-methylpyrrolidinium bis((trifluoromethanesulfonyl imide
PYR_13_TFSI	N-propyl-n-methylpyrrolidinium bis((trifluoromethanesulfonyl imide
PVdF-co-HFP	Poly vinylidenefluoride –co-hexafluoropropylene
PP_13_TFSI	N-propyl-n-methylphosphonium bis((trifluoromethanesulfonyl imide
PYRNO_3_	Pyrrolidinium nitrate
PC	Propylene carbonate
P_111i4_FSI	Trimethylisobutylphosphonium bis(fluorosulfonyl) amide
PYR_24_FSI	*N*-butyl-*N*-ethylpyrrolidinium bis(fluorosulfonyl) amide
PEO; PMMA	Poly (ethylene oxide); Poly (methylmethacrylate)
PSt-b-PMMA-b-Pst	polystyrene-block-poly methyl methacrylate-block-polystyrene
PAN	Poly (acrylonitrile)
PANI	Polyaniline
PDMS	Polydimethylsiloxane
Pip_13_TFSI	1-methyl-1-propylpiperidinium bis(trifluoro-methane sulfonyl)imide
PEGMA; BEMA	Polyethylene glycol methacrylate; Butyl methyl methacrylate
PEDOT	Poly(3,4-ethylenedioxythiophene)
PoILs	Polymeric Ionic liquids
PVA	Polyvinyl Alcohol
QATFSI	butyl-trimethyl ammonium bis(trifluoromethanesulfonyl)imide
RTILs	Room temperature Ionic liquids
RGO/rGO	Reduced graphene oxide
SCs/SC	Supercapacitors/Supercapacitor
SEI	Solid electrolyte interface
SWCNT	Single-walled carbon nanotubes
SPEs	Solid polymer electrolytes
TMHATFSI	Trimethylhexylammonium bis(trifluoro-methane sulfonyl)imide
TEOS	Tetra ethoxy silane
TFSI	Bis(trifluoromethanesulfonyl imide)
TGPEs	ternary gel polymer electrolytes
VC	vinylene carbonate
1g_13_TFSI	1-methyl-3-propylguanivdinium bis(trifluoromethanesulfonyl) amide

## References

[1] Forsyth M., Porcarelli L., Wang X., Goujon N., Mecerreyes D. (2019). Innovative electrolytes based on ionic liquids and polymers for next-generation solid-state batteries. Acc. Chem. Res..

[2] Vikraman D., Patil S.A., Hussain S., Mengal N., Jeong S.H., Jung J., Park H.J., Kim H.-S., Kim H.-S. (2019). Construction of dye-sensitized solar cells using wet chemical route synthesized MoSe2 counter electrode. J. Ind. Eng. Chem..

[3] Kim J.-H., Jung M.-J., Kim M.-J., Lee Y.-S. (2018). Electrochemical performances of lithium and sodium ion batteries based on carbon materials. J. Ind. Eng. Chem..

[4] Eshetu G.G., Armand M., Scrosati B., Passerini S. (2014). Energy Storage Materials Synthesized from Ionic Liquids. Angew. Chem. Int. Ed..

[5] Karuppasamy K., Prasanna K., Ilango P.R., Vikraman D., Bose R., Alfantazi A., Kim H.-S. (2019). Biopolymer phytagel-derived porous nanocarbon as efficient electrode material for high-performance symmetric solid-state supercapacitors. J. Ind. Eng. Chem..

[6] Iamprasertkun P., Krittayavathananon A., Sawangphruk M. (2016). N-doped reduced graphene oxide aerogel coated on carboxyl-modified carbon fiber paper for high-performance ionic-liquid supercapacitors. Carbon N. Y..

[7] Tsao C.-H., Su H.-M., Huang H.-T., Kuo P.-L., Teng H. (2019). Immobilized cation functional gel polymer electrolytes with high lithium transference number for lithium ion batteries. J. Membr. Sci..

[8] Navalpotro P., Palma J., Anderson M., Marcilla R. (2016). High performance hybrid supercapacitors by using para-Benzoquinone ionic liquid redox electrolyte. J. Power Sources.

[9] Yu G., Xie X., Pan L., Bao Z., Cui Y. (2013). Hybrid nanostructured materials for high-performance electrochemical capacitors. Nano Energy.

[10] Gu W., Yushin G. (2014). Review of nanostructured carbon materials for electrochemical capacitor applications: advantages and limitations of activated carbon, carbide-derived carbon, zeolite-templated carbon, carbon aerogels, carbon nanotubes, onion-like carbon, and graphene. Wiley Interdiscip. Rev. Energy Environ..

[11] Osada I., de Vries H., Scrosati B., Passerini S. (2016). Ionic-Liquid-Based Polymer Electrolytes for Battery Applications. Angew. Chem. Int. Ed..

[12] Navarra M.A. (2013). Ionic liquids as safe electrolyte components for Li-metal and Li-ion batteries. MRS Bull..

[13] Wang Y., Song Y., Xia Y. (2016). Electrochemical capacitors: mechanism, materials, systems, characterization and applications. Chem. Soc. Rev..

[14] Liew C.W., Ramesh S., Arof A.K. (2016). Design, Investigation of ionic liquid-doped ion conducting polymer electrolytes for carbon-based electric double layer capacitors (EDLCs). Mater. Des..

[15] Demarconnay L., Calvo E.G., Timperman L., Anouti M., Lemordant D., Raymundo-Piñero E., Arenillas A., Menéndez J.A., Béguin F. (2013). Optimizing the performance of supercapacitors based on carbon electrodes and protic ionic liquids as electrolytes. Electrochim. Acta.

[16] Wang A., Liu X., Wang S., Chen J., Xu H., Xing Q., Zhang L. (2018). Polymeric ionic liquid enhanced all-solid-state electrolyte membrane for high-performance lithium-ion batteries. Electrochim. Acta.

[17] Eshetu G.G., Armand M., Ohno H., Scrosati B., Passerini S. (2016). Ionic liquids as tailored media for the synthesis and processing of energy conversion materials. Energy Environ. Sci..

[18] Wang S., Shi Q.X., Ye Y.S., Xue Y., Wang Y., Peng H.Y., Xie X.L., Mai Y.W. (2017). Constructing desirable ion-conducting channels within ionic liquid-based composite polymer electrolytes by using polymeric ionic liquid-functionalized 2D mesoporous silica nanoplates. Nano Energy.

[19] Onuma T., Hosono E., Takenouchi M., Sakuda J., Kajiyama S., Yoshio M., Kato T. (2018). Noncovalent approach to liquid-crystalline ion conductors: high-rate performances and room-temperature operation for Li-ion batteries. ACS Omega.

[20] Kim G.T., Jeong S.S., Xue M.Z., Balducci A., Winter M., Passerini S., Alessandrini F., Appetecchi G.B. (2012). Development of ionic liquid-based lithium battery prototypes. J. Power Sources.

[21] Navarra M.A., Manzi J., Lombardo L., Panero S., Scrosati B. (2011). Ionic Liquid-Based Membranes as Electrolytes for Advanced Lithium Polymer Batteries. ChemSusChem.

[22] Yoon H., Howlett P.C., Best A.S., Forsyth M., MacFarlane D.R. (2013). Fast charge/discharge of Li metal batteries using an ionic liquid electrolyte. J. Electrochem. Soc..

[23] Macfarlane D.R., Tachikawa N., Forsyth M., Pringle J.M., Howlett P.C., Elliott G.D., Davis J.H., Watanabe M., Simon P., Angell C.A. (2014). Energy applications of ionic liquids. Energy Environ. Sci..

[24] Noor S.A.M., Bayley P.M., Forsyth M., MacFarlane D.R. (2013). Ionogels based on ionic liquids as potential highly conductive solid state electrolytes. Electrochim. Acta.

[25] MacFarlane D.R., Forsyth M., Howlett P.C., Kar M., Passerini S., Pringle J.M., Ohno H., Watanabe M., Yan F., Zheng W. (2016). Ionic liquids and their solid-state analogues as materials for energy generation and storage. Nat. Rev. Mater..

[26] Varela J.C., Sankar K., Hino A., Lin X., Chang W.-s., Coker D., Grinstaff M. (2018). Piperidinium ionic liquids as electrolyte solvents for sustained high temperature supercapacitor operation. Chem. Commun..

[27] Schaefer J.L., Lu Y., Moganty S.S., Agarwal P., Jayaprakash N., Archer L.A. (2012). Electrolytes for high-energy lithium batteries. Appl. Nanosci..

[28] Mousavi M.P.S., Wilson B.E., Kashefolgheta S., Anderson E.L., He S., Bühlmann P., Stein A. (2016). Stein, interfaces, Ionic liquids as electrolytes for electrochemical double-layer capacitors: structures that optimize specific energy. ACS Appl. Mater. Interfaces.

[29] Srour H., Rouault H., Santini C. (2012). Imidazolium based ionic liquid electrolytes for Li-ion secondary batteries based on graphite and LiFePO_4_. J. Electrochem. Soc..

[30] Wu T.-Y., Chen B.-K., Hao L., Lin K.-F., Sun I.-W. (2011). Thermophysical properties of a room temperature ionic liquid (1-methyl-3-pentyl-imidazolium hexafluorophosphate) with poly (ethylene glycol). J. Taiwan Inst. Chem. Eng..

[31] Ferrari S., Quartarone E., Tomasi C., Ravelli D., Protti S., Fagnoni M., Mustarelli P. (2013). Alkoxy substituted imidazolium-based ionic liquids as electrolytes for lithium batteries. J. Power Sources.

[32] Weber R.L., Ye Y., Banik S.M., Elabd Y.A., Hickner M.A., Mahanthappa M.K. (2011). Thermal and ion transport properties of hydrophilic and hydrophobic polymerized styrenic imidazolium ionic liquids. J. Polym. Sci. Part B Polym. Phys..

[33] Gerbaldi C., Nair J.R., Ferrari S., Chiappone A., Meligrana G., Zanarini S., Mustarelli P., Penazzi N., Bongiovanni R. (2012). New electrolyte membranes for Li-based cells: Methacrylic polymers encompassing pyrrolidinium-based ionic liquid by single step photo-polymerisation. J. Memb. Sci..

[34] Pitawala J., Navarra M.A., Scrosati B., Jacobsson P., Matic A. (2014). Structure and properties of Li-ion conducting polymer gel electrolytes based on ionic liquids of the pyrrolidinium cation and the bis (trifluoromethanesulfonyl) imide anion. J. Power Sources.

[35] Yang B., Li C., Zhou J., Liu J., Zhang Q. (2014). Pyrrolidinium-based ionic liquid electrolyte with organic additive and LiTFSI for high-safety lithium-ion batteries. Electrochim. Acta.

[36] Ivanov S., Cheng L., Wulfmeier H., Albrecht D., Fritze H., Bunda A. (2013). Electrochemical behavior of anodically obtained titania nanotubes in organic carbonate and ionic liquid based Li ion containing electrolytes. Electrochim. Acta.

[37] Chakrapani V., Rusli F., Filler M.A., Kohl P.A. (2011). Quaternary ammonium ionic liquid electrolyte for a silicon nanowire-based lithium ion battery. J. Phys. Chem. C.

[38] Fisher A.S., Khalid M.B., Widstrom M., Kofinas P. (2012). Anion effects on solid polymer electrolytes containing sulfur based ionic liquid for lithium batteries. J. Electrochem. Soc..

[39] Li M., Yang L., Fang S., Dong S. (2011). Novel polymeric ionic liquid membranes as solid polymer electrolytes with high ionic conductivity at moderate temperature. J. Memb. Sci..

[40] Chaurasia S.K., Singh R.K., Chandra S. (2013). Thermal stability, complexing behavior, and ionic transport of polymeric gel membranes based on polymer PVdF-HFP and ionic liquid,[BMIM][BF4]. J. Phys. Chem. B.

[41] Sarangika H.N.M., Dissanayake M.A.K.L., Senadeera G.K.R., Rathnayake R.R.D.V., Pitawala H.M.J.C. (2017). Polyethylene oxide and ionic liquid-based solid polymer electrolyte for rechargeable magnesium batteries. Ionics (Kiel).

[42] Kirchhöfer M., von Zamory J., Paillard E., Passerini S. (2014). Separators for Li-ion and Li-Metal battery including ionic liquid based electrolytes based on the TFSI− and FSI− anions. Int. J. Mol. Sci..

[43] Reiter J., Nadherna M. (2012). N-Allyl-N-methylpiperidinium bis (trifluoromethanesulfonyl) imide—A film forming ionic liquid for graphite anode of Li-ion batteries. Electrochim. Acta.

[44] Karuppasamy K., Reddy P.A., Srinivas G., Sharma R., Tewari A., Kumar G.H., Gupta D. (2016). An efficient way to achieve high ionic conductivity and electrochemical stability of safer nonaflate anion-based ionic liquid gel polymer electrolytes (ILGPEs) for rechargeable lithium ion batteries. J. Solid State Electrochem..

[45] Karuppasamy K., Reddy P.A., Srinivas G., Tewari A., Sharma R., Shajan X.S., Gupta D. (2016). Electrochemical and cycling performances of novel nonafluorobutanesulfonate (nonaflate) ionic liquid based ternary gel polymer electrolyte membranes for rechargeable lithium ion batteries. J. Memb. Sci..

[46] Karuppasamy K., Prasanna K., Kim D., Kang Y.H., Rhee H.W. (2017). Headway in rhodanide anion based ternary gel polymer electrolytes (TILGPEs) for applications in rechargeable lithium ion batteries: an efficient route to achieve high electrochemical and cycling performances. RSC Adv..

[47] Tiruye G.A., Muñoz-Torrero D., Palma J., Anderson M., Marcilla R. (2015). All-solid state supercapacitors operating at 3.5 V by using ionic liquid based polymer electrolytes. J. Power Sources.

[48] Plylahan N., Kerner M., Lim D.H., Matic A., Johansson P. (2016). Ionic liquid and hybrid ionic liquid/organic electrolytes for high temperature lithium-ion battery application. Electrochim. Acta.

[49] Unemoto A., Ogawa H., Gambe Y., Honma I. (2014). Development of lithium-sulfur batteries using room temperature ionic liquid-based quasi-solid-state electrolytes. Electrochim. Acta.

[50] Hallinan D.T., Balsara N.P. (2013). Polymer Electrolytes. Annu. Rev. Mater. Sci..

[51] Singh V.K., Singh R.K. (2015). Development of ion conducting polymer gel electrolyte membranes based on polymer PVdF-HFP, BMIMTFSI ionic liquid and the Li-salt with improved electrical, thermal and structural properties. J. Mater. Chem. C.

[52] Kühnel R.S., Böckenfeld N., Passerini S., Winter M., Balducci A. (2011). Mixtures of ionic liquid and organic carbonate as electrolyte with improved safety and performance for rechargeable lithium batteries. Electrochim. Acta.

[53] Xiang H.F., Yin B., Wang H., Lin H.W., Ge X.W., Xie S., Chen C.H. (2010). Improving electrochemical properties of room temperature ionic liquid (RTIL) based electrolyte for Li-ion batteries. Electrochim. Acta.

[54] Theivaprakasam S., MacFarlane D.R., Mitra S. (2015). Electrochemical studies of N-Methyl N-Propyl Pyrrolidinium bis (trifluoromethanesulfonyl) imide ionic liquid mixtures with conventional electrolytes in LiFePO_4_/Li cells. Electrochim. Acta.

[55] Appetecchi G.B., Kim G.T., Montanino M., Alessandrini F., Passerini S. (2011). Room temperature lithium polymer batteries based on ionic liquids. J. Power Sources.

[56] Kim G.T., Appetecchi G.B., Carewska M., Joost M., Balducci A., Winter M., Passerini S. (2010). UV cross-linked, lithium-conducting ternary polymer electrolytes containing ionic liquids. J. Power Sources.

[57] Yun Y.S., Kim J.H., Lee S.Y., Shim E.G., Kim D.W. (2011). Cycling performance and thermal stability of lithium polymer cells assembled with ionic liquid-containing gel polymer electrolytes. J. Power Sources.

[58] Hofmann A., Schulz M., Hanemann T. (2013). Gel electrolytes based on ionic liquids for advanced lithium polymer batteries. Electrochim. Acta.

[59] Amereller M., Schedlbauer T., Moosbauer D., Schreiner C., Stock C., Wudy F., Zugmann S., Hammer H., Maurer A., Gschwind R.M. (2014). Electrolytes for lithium and lithium ion batteries: From synthesis of novel lithium borates and ionic liquids to development of novel measurement methods. Prog. Solid State Chem..

[60] Böckenfeld N., Kühnel R.S., Passerini S., Winter M., Balducci A. (2011). Composite LiFePO_4_/AC high rate performance electrodes for Li-ion capacitors. J. Power Sources.

[61] Rui X.H., Jin Y., Feng X.Y., Zhang L.C., Chen C.H. (2011). A comparative study on the low-temperature performance of LiFePO_4_/C and Li3V2 (PO4) 3/C cathodes for lithium-ion batteries. J. Power Sources.

[62] Kramer E., Schedlbauer T., Hoffmann B., Terborg L., Nowak S., Gores H.J., Passerini S., Winter M. (2012). Mechanism of anodic dissolution of the aluminum current collector in 1 M LiTFSI EC: DEC 3: 7 in rechargeable lithium batteries. J. Electrochem. Soc..

[63] Hofmann A., Merklein L., Schulz M., Hanemann T. (2014). Low-flammable electrolytes with fluoroethylene carbonate based solvent mixtures and lithium bis (trifluoromethanesulfonyl) imide for lithium-ion batteries. Electrochim. Acta.

[64] Bockenfeld N., Willeke M., Pires J., Anouti M., Balducci A. (2013). On the use of lithium iron phosphate in combination with protic ionic liquid-based electrolytes. J. Electrochem. Soc..

[65] Timperman L., Galiano H., Lemordant D., Anouti M. (2011). Phosphonium-based protic ionic liquid as electrolyte for carbon-based supercapacitors. Electrochem. Commun..

[66] Suryanto B.H.R., Gunawan C.A., Lu X., Zhao C. (2012). Tuning the electrodeposition parameters of silver to yield micro/nano structures from room temperature protic ionic liquids. Electrochim. Acta.

[67] Mattedi S., Carvalho P.J., Coutinho J.A.P., Alvarez V.H., Iglesias M. (2011). High pressure CO2 solubility in N-methyl-2-hydroxyethylammonium protic ionic liquids. J. Supercrit. Fluids.

[68] Tachikawa N., Yamauchi K., Takashima E., Park J.-W., Dokko K., Watanabe M. (2011). Reversibility of electrochemical reactions of sulfur supported on inverse opal carbon in glyme–Li salt molten complex electrolytes. Chem. Commun..

[69] Wang L., Byon H.R. (2013). N-Methyl-N-propylpiperidinium bis (trifluoromethanesulfonyl) imide-based organic electrolyte for high performance lithium–sulfur batteries. J. Power Sources.

[70] Nelson J., Misra S., Yang Y., Jackson A., Liu Y., Wang H., Dai H., Andrews J.C., Cui Y., Toney M.F. (2012). In operando X-ray diffraction and transmission X-ray microscopy of lithium sulfur batteries. J. Am. Chem. Soc..

[71] Lin X., Salari M., Mohana L., Arava R., Ajayan P.M., Grinstaff M.W. (2016). High temperature electrical energy storage: advances, challenges, and frontiers. Chem. Soc. Rev..

[72] Simon P., Gogotsi Y. (2008). Materials for electrochemical capacitors. Nat. Mater..

[73] Kerner M., Plylahan N., Scheers J., Johansson P. (2015). Ionic liquid based lithium battery electrolytes: fundamental benefits of utilising both TFSI and FSI anions?. Phys. Chem. Chem. Phys..

[74] Wang S.H., Lin Y.Y., Teng C.Y., Chen Y.M., Kuo P.L., Lee Y.L., Hsieh C.T., Teng H. (2016). Immobilization of anions on polymer matrices for gel electrolytes with high conductivity and stability in lithium ion batteries. ACS Appl. Mater. Interfaces.

[75] Hernandez G., Isik M., Mantione D., Pendashteh A., Navalpotro P., Shanmukaraj D., Marcilla R., Mecerreyes D. (2017). Redox-active poly (ionic liquid) s as active materials for energy storage applications. J. Mater. Chem. A.

[76] Liao C., Guo B., Sun X.G., Dai S. (2015). Synergistic effects of mixing sulfone and ionic liquid as safe electrolytes for lithium sulfur batteries. ChemSusChem.

[77] Gao X.W., Feng C.Q., Chou S.L., Wang J.Z., Sun J.Z., Forsyth M., MacFarlane D.R., Liu H.K. (2013). LiNi0. 5Mn1. 5O4 spinel cathode using room temperature ionic liquid as electrolyte. Electrochim. Acta.

[78] Li S., Li B., Xu X., Shi X., Zhao Y., Mao L., Cui X. (2012). Electrochemical performances of two kinds of electrolytes based on lithium bis (oxalate) borate and sulfolane for advanced lithium ion batteries. J. Power Sources.

[79] Hofmann A., Migeot M., Arens L., Hanemann T. (2016). Investigation of Ternary Mixtures Containing 1-Ethyl-3-methylimidazolium Bis(trifluoromethanesulfonyl)azanide, Ethylene Carbonate and Lithium Bis(trifluoromethanesulfonyl)azanide. Int. J. Mol. Sci..

[80] Castiglione F., Ragg E., Mele A., Appetecchi G.B., Montanino M., Passerini S. (2011). Molecular environment and enhanced diffusivity of Li+ ions in lithium-salt-doped ionic liquid electrolytes. J. Phys. Chem. Lett..

[81] Bi S., Banda H., Chen M., Niu L., Chen M., Wu T., Wang J., Wang R., Feng J., Chen T. (2020). Molecular understanding of charge storage and charging dynamics in supercapacitors with MOF electrodes and ionic liquid electrolytes. Nat. Mater..

[82] Dilasari B., Jung Y., Kim G., Kwon K. (2016). Effect of Cation Structure on Electrochemical Behavior of Lithium in [NTf2]-based Ionic Liquids. ACS Sustain. Chem. Eng..

[83] Wang M., Shan Z., Tian J., Yang K., Liu X., Liu H., Zhu K. (2013). Mixtures of unsaturated imidazolium based ionic liquid and organic carbonate as electrolyte for Li-ion batteries. Electrochim. Acta.

[84] Francis C.F.J., Kyratzis I.L., Best A.S. (2020). Lithium-Ion Battery Separators for Ionic-Liquid Electrolytes: A Review. Adv. Mater..

[85] Hofmann A., Schulz M., Indris S., Heinzmann R., Hanemann T. (2014). Mixtures of ionic liquid and sulfolane as electrolytes for Li-ion batteries. Electrochim. Acta.

[86] Montanino M., Moreno M., Carewska M., Maresca G., Simonetti E., Presti R.L., Alessandrini F., Appetecchi G.B. (2014). Mixed organic compound-ionic liquid electrolytes for lithium battery electrolyte systems. J. Power Sources.

[87] Gao X., Mariani A., Jeong S., Liu X., Dou X., Ding M., Moretti A., Passerini S. (2019). Prototype rechargeable magnesium batteries using ionic liquid electrolytes. J. Power Sources.

[88] Ababtain K., Babu G., Lin X., Rodrigues M.T.F., Gullapalli H., Ajayan P.M., Grinstaff M.W., Arava L.M.R. (2016). Ionic liquid–organic carbonate electrolyte blends to stabilize silicon electrodes for extending lithium ion battery operability to 100 C. ACS Appl. Mater. Interfaces.

[89] Cao X., He X., Wang J., Liu H., Röser S., Rad B.R., Evertz M., Streipert B., Li J., Wagner R. (2016). High voltage LiNi0. 5Mn1. 5O4/Li4Ti5O12 lithium ion cells at elevated temperatures: Carbonate-versus ionic liquid-based electrolytes. ACS Appl. Mater. Interfaces.

[90] Usui H., Yamamoto Y., Yoshiyama K., Itoh T., Sakaguchi H. (2011). Application of electrolyte using novel ionic liquid to Si thick film anode of Li-ion battery. J. Power Sources.

[91] Liao Y., Sun C., Hu S., Li W. (2013). Anti-thermal shrinkage nanoparticles/polymer and ionic liquid based gel polymer electrolyte for lithium ion battery. Electrochim. Acta.

[92] Lu Y., Moganty S.S., Schaefer J.L., Archer L.A. (2012). Ionic liquid-nanoparticle hybrid electrolytes. J. Mater. Chem..

[93] Lu Y., Korf K., Kambe Y., Tu Z., Archer L.A. (2014). Ionic-liquid–nanoparticle hybrid electrolytes: applications in lithium metal batteries. Angew. Chem. Int. Ed..

[94] Fujii K., Hamano H., Doi H., Song X., Tsuzuki S., Hayamizu K., Seki S., Kameda Y., Dokko K., Watanabe M. (2013). Unusual Li+ ion solvation structure in bis (fluorosulfonyl) amide based ionic liquid. J. Phys. Chem. C.

[95] Umebayashi Y., Hamano H., Seki S., Minofar B., Fujii K., Hayamizu K., Tsuzuki S., Kameda Y., Kohara S., Watanabe M. (2011). Liquid structure of and Li+ ion solvation in bis (trifluoromethanesulfonyl) amide based ionic liquids composed of 1-ethyl-3-methylimidazolium and N-methyl-N-propylpyrrolidinium cations. J. Phys. Chem. B.

[96] Grande L., von Zamory J., Koch S.L., Kalhoff J., Paillard E., Passerini S. (2015). Homogeneous lithium electrodeposition with pyrrolidinium-based ionic liquid electrolytes. ACS Appl. Mater. Interfaces.

[97] Forsyth M., Girard G.M.A., Basile A., Hilder M., MacFarlane D.R., Chen F., Howlett P.C. (2016). Inorganic-organic ionic liquid electrolytes enabling high energy-density metal electrodes for energy storage. Electrochim. Acta.

[98] Wu F., Chen N., Chen R., Zhu Q., Qian J., Li L. (2016). “Liquid-in-solid” and “solid-in-liquid” electrolytes with high rate capacity and long cycling life for lithium-ion batteries. Chem. Mater..

[99] Deshpande A., Kariyawasam L., Dutta P., Banerjee S. (2013). Enhancement of lithium ion mobility in ionic liquid electrolytes in presence of additives. J. Phys. Chem. C.

[100] Moreno J.S., Deguchi Y., Panero S., Scrosati B., Ohno H., Simonetti E., Appetecchi G.B. (2016). N-Alkyl-N-ethylpyrrolidinium cation-based ionic liquid electrolytes for safer lithium battery systems. Electrochim. Acta.

[101] Forsyth S.A., Pringle J.M., MacFarlane D.R. (2004). Ionic liquids—an overview. Aust. J. Chem..

[102] Moreno M., Simonetti E., Appetecchi G.B., Carewska M., Montanino M., Kim G.-T., Loeffler N., Passerini S. (2017). Ionic liquid electrolytes for safer lithium batteries I. Investigation around optimal formulation. J. Electrochem. Soc..

[103] Guo Q., Han Y., Wang H., Hong X., Zheng C., Liu S., Xie K., Scrosati B., Garche J., Etacheri V. (2016). Safer lithium metal battery based on advanced ionic liquid gel polymer nonflammable electrolytes. RSC Adv..

[104] Sim L.N., Majid S.R., Arof A.K. (2014). Effects of 1–butyl–3–methyl imidazolium trifluoromethanesulfonate ionic liquid in poly(ethyl methacrylate)/poly(vinylidenefluoride–co–hexafluoropropylene) blend based polymer electrolyte system. Electrochim. Acta.

[105] Appetecchi G.B., Montanino M., Passerini S. (2012). Ionic Liquid-Based Electrolytes for High Energy, Safer Lithium Batteries. Ionic Liquids: Science and Applications.

[106] Kubota K., Matsumoto H. (2013). Investigation of an Intermediate Temperature Molten Lithium Salt Based on Fluorosulfonyl(trifluoromethylsulfonyl)amide as a Solvent-Free Lithium Battery Electrolyte. J. Phys. Chem. C.

[107] Placke T., Fromm O., Lux S.F., Bieker P., Rothermel S., Meyer H.-W., Passerini S., Winter M. (2012). Reversible Intercalation of Bis(trifluoromethanesulfonyl)imide Anions from an Ionic Liquid Electrolyte into Graphite for High Performance Dual-Ion Cells. J. Electrochem. Soc..

[108] Lesch V., Jeremias S., Moretti A., Passerini S., Heuer A., Borodin O. (2014). A Combined Theoretical and Experimental Study of the Influence of Different Anion Ratios on Lithium Ion Dynamics in Ionic Liquids. J. Phys. Chem. B.

[109] Weingarth D., Czekaj I., Fei Z., Foelske-Schmitz A., Dyson P.J., Wokaun A., Kotz R. (2012). Electrochemical Stability of Imidazolium Based Ionic Liquids Containing Cyano Groups in the Anion: A Cyclic Voltammetry, XPS and DFT Study. J. Electrochem. Soc..

[110] Ueno K., Yoshida K., Tsuchiya M., Tachikawa N., Dokko K., Watanabe M. (2012). Glyme–Lithium Salt Equimolar Molten Mixtures: Concentrated Solutions or Solvate Ionic Liquids?. J. Phys. Chem. B.

[111] Zhang C., Ueno K., Yamazaki A., Yoshida K., Moon H., Mandai T., Umebayashi Y., Dokko K., Watanabe M. (2014). Chelate Effects in Glyme/Lithium Bis(trifluoromethanesulfonyl)amide Solvate Ionic Liquids. I. Stability of Solvate Cations and Correlation with Electrolyte Properties. J. Phys. Chem. B.

[112] Kuo P.L., Tsao C.H., Hsu C.H., Chen S.T., Hsu H.M. (2016). A new strategy for preparing oligomeric ionic liquid gel polymer electrolytes for high-performance and nonflammable lithium ion batteries. J. Memb. Sci..

[113] Ueno K., Murai J., Moon H., Dokko K., Watanabe M. (2017). A Design Approach to Lithium-Ion Battery Electrolyte Based on Diluted Solvate Ionic Liquids. J. Electrochem. Soc..

[114] Liu C., Ma X., Xu F., Zheng L., Zhang H., Feng W., Huang X., Armand M., Nie J., Chen H. (2014). Ionic liquid electrolyte of lithium bis(fluorosulfonyl)imide/N-methyl-N-propylpiperidinium bis(fluorosulfonyl)imide for Li/natural graphite cells: Effect of concentration of lithium salt on the physicochemical and electrochemical properties. Electrochim. Acta.

[115] Schedlbauer T., Krüger S., Schmitz R., Schmitz R.W., Schreiner C., Gores H.J., Passerini S., Winter M. (2013). Lithium difluoro(oxalato)borate: A promising salt for lithium metal based secondary batteries?. Electrochim. Acta.

[116] Appetecchi G.B., Montanino M., Balducci A., Lux S.F., Winter M., Passerini S. (2012). Lithium insertion in graphite from ternary ionic liquid-lithium salt electrolytes I. Electrochem. Charact. Electrolytes J. Power Sources.

[117] Mun J., Yim T., Park K., Ryu J.H., Kim Y.G., Oh S.M. (2011). Surface Film Formation on LiNi_0.5_Mn_1.5_O_4_ Electrode in an Ionic Liquid Solvent at Elevated Temperature. J. Electrochem. Soc..

[118] Geiculescu O.E., Desmarteau D.D., Creager S.E., Haik O., Hirshberg D., Shilina Y., Zinigrad E., Levi M.D., Aurbach D., Halalay I.C. (2016). Novel binary deep eutectic electrolytes for rechargeable Li-ion batteries based on mixtures of alkyl sulfonamides and lithium perfluoroalkylsulfonimide salts. J. Power Sources.

[119] Hu P., Duan Y., Hu D., Qin B., Zhang J., Wang Q., Liu Z., Cui G., Chen L. (2015). Rigid–Flexible Coupling High Ionic Conductivity Polymer Electrolyte for an Enhanced Performance of LiMn_2_O_4_/Graphite Battery at Elevated Temperature. ACS Appl. Mater. Interfaces.

[120] Karuppasamy K., Rhee H.W., Reddy P.A., Gupta D., Mitu L., Polu A.R., Shajan X.S. (2016). Ionic liquid incorporated nanocomposite polymer electrolytes for rechargeable lithium ion battery: A way to achieve improved electrochemical and interfacial properties. J. Ind. Eng. Chem..

[121] Li H., Chen Y.M., Ma X.T., Shi J.L., Zhu B.K., Zhu L.P. (2011). Gel polymer electrolytes based on active PVDF separator for lithium ion battery. I: Preparation and property of PVDF/poly(dimethylsiloxane) blending membrane. J. Memb. Sci..

[122] Tamilarasan P., Ramaprabhu S. (2013). Graphene based all-solid-state supercapacitors with ionic liquid incorporated polyacrylonitrile electrolyte. Energy.

[123] Li Y., Wong K.W., Dou Q., Ng K.M. (2016). A single-ion conducting and shear-thinning polymer electrolyte based on ionic liquid-decorated PMMA nanoparticles for lithium-metal batteries. J. Mater. Chem. A.

[124] Rao M., Geng X., Liao Y., Hu S., Li W. (2012). Preparation and performance of gel polymer electrolyte based on electrospun polymer membrane and ionic liquid for lithium ion battery. J. Memb. Sci..

[125] Kitazawa Y., Iwata K., Imaizumi S., Ahn H., Kim S.Y., Ueno K., Park M.J., Watanabe M. (2014). Gelation of Solvate Ionic Liquid by Self-Assembly of Block Copolymer and Characterization as Polymer Electrolyte. Macromolecules.

[126] Patel M., Chandrappa K.G., Bhattacharyya A.J. (2010). Increasing ionic conductivity of polymer–sodium salt complex by addition of a non-ionic plastic crystal. Solid State Ionics.

[127] Patel M., Gnanavel M., Bhattacharyya A.J. (2011). Utilizing an ionic liquid for synthesizing a soft matter polymer “gel” electrolyte for high rate capability lithium-ion batteries. J. Mater. Chem..

[128] Lewandowski A., Swiderska-Mocek A., Waliszewski L. (2013). Li+ conducting polymer electrolyte based on ionic liquid for lithium and lithium-ion batteries. Electrochim. Acta.

[129] Carol P., Ramakrishnan P., John B., Cheruvally G. (2011). Preparation and characterization of electrospun poly(acrylonitrile) fibrous membrane based gel polymer electrolytes for lithium-ion batteries. J. Power Sources.

[130] Liao C., Sun X.-G., Dai S. (2013). Crosslinked gel polymer electrolytes based on polyethylene glycol methacrylate and ionic liquid for lithium ion battery applications. Electrochim. Acta.

[131] Zhou D., Zhou R., Chen C., Yee W.A., Kong J., Ding G., Lu X. (2013). Non-Volatile Polymer Electrolyte Based on Poly(propylene carbonate), Ionic Liquid, and Lithium Perchlorate for Electrochromic Devices. J. Phys. Chem. B.

[132] Ravi M., Song S., Wang J., Wang T., Nadimicherla R. (2016). Ionic liquid incorporated biodegradable gel polymer electrolyte for lithium ion battery applications. J. Mater. Sci. Mater. Electron..

[133] Stepniak I., Andrzejewska E., Dembna A., Galinski M. (2014). Characterization and application of N-methyl-N-propylpiperidinium bis(trifluoromethanesulfonyl)imide ionic liquid–based gel polymer electrolyte prepared in situ by photopolymerization method in lithium ion batteries. Electrochim. Acta.

[134] Li W., Li Z., Yang C., Xiao Q., Lei G., Ding Y. (2016). A capsule-type gelled polymer electrolyte for rechargeable lithium batteries. RSC Adv..

[135] Karuppasamy K., Kim H.-S., Kim D., Vikraman D., Prasanna K., Kathalingam A., Sharma R., Rhee H.W. (2017). An enhanced electrochemical and cycling properties of novel boronic Ionic liquid based ternary gel polymer electrolytes for rechargeable Li/LiCoO_2_ cells. Sci. Rep..

[136] Li M., Yang L., Fang S., Dong S., Hirano S.I., Tachibana K. (2011). Polymer electrolytes containing guanidinium-based polymeric ionic liquids for rechargeable lithium batteries. J. Power Sources.

[137] Choi N.-S., Koo B., Yeon J.-T., Lee K.T., Kim D.-W. (2011). Effect of a novel amphipathic ionic liquid on lithium deposition in gel polymer electrolytes. Electrochim. Acta.

[138] Park J.H., Moon J., Han S., Park S., Lim J.W., Yun D.J., Kim D.Y., Park K., Son I.H. (2017). Formation of Stable Solid–Electrolyte Interphase Layer on Few-Layer Graphene-Coated Silicon Nanoparticles for High-Capacity Li-Ion Battery Anodes. J. Phys. Chem. C.

[139] Chinnam P.R., Zhang H., Wunder S.L. (2015). Blends of Pegylated Polyoctahedralsilsesquioxanes (POSS-PEG) and Methyl Cellulose as Solid Polymer Electrolytes for Lithium Batteries. Electrochim. Acta.

[140] Isa K.B., Osman Z., Arof A.K., Othman L., Zainol N.H., Samin S.M., Chong W.G., Kamarulzaman N. (2014). Lithium ion conduction and ion–polymer interaction in PVdF-HFP based gel polymer electrolytes. Solid State Ionics.

[141] Karuppasamy K., Kim D., Kang Y.H., Prasanna K., Rhee H.W. (2017). Improved electrochemical, mechanical and transport properties of novel lithium bisnonafluoro-1-butanesulfonimidate (LiBNFSI) based solid polymer electrolytes for rechargeable lithium ion batteries. J. Ind. Eng. Chem..

[142] Karuppasamy K., Antony R., Alwin S., Balakumar S., Shajan X.S. (2015). A Review on PEO Based Solid Polymer Electrolytes (SPEs) Complexed with LiX (X=Tf, BOB) for Rechargeable Lithium Ion Batteries. Mater. Sci. Forum.

[143] Kumar Y., Hashmi S.A., Pandey G.P. (2011). Lithium ion transport and ion–polymer interaction in PEO based polymer electrolyte plasticized with ionic liquid. Solid State Ionics.

[144] Diddens D., Heuer A. (2013). Lithium Ion Transport Mechanism in Ternary Polymer Electrolyte-Ionic Liquid Mixtures: A Molecular Dynamics Simulation Study. ACS Macro Lett..

[145] Karuppasamy K., Thanikaikarasan S., Antony R., Balakumar S., Shajan X.S. (2012). Effect of nanochitosan on electrochemical, interfacial and thermal properties of composite solid polymer electrolytes. Ionics (Kiel).

[146] Karuppasamy K., Vani C.V., Nichelson A., Balakumar S., Shajan X.S. (2013). Effect of nanochitosan and succinonitrile on the AC ionic conductivity of plasticized nanocomposite solid polymer electrolytes (PNCSPE). AIP Conf. Proc..

[147] Karuppasamy K., Antony R., Thanikaikarasan S., Balakumar S., Shajan X.S. (2013). Combined effect of nanochitosan and succinonitrile on structural, mechanical, thermal, and electrochemical properties of plasticized nanocomposite polymer electrolytes (PNCPE) for lithium batteries. Ionics (Kiel).

[148] Chaudoy V., Ghamouss F., Luais E., Tran-Van F. (2016). Cross-Linked Polymer Electrolytes for Li-Based Batteries: From Solid to Gel Electrolytes. Ind. Eng. Chem. Res..

[149] Karmakar A., Ghosh A. (2011). Ac conductivity and relaxation in CdO doped poly ethylene oxide-LiI nanocomposite electrolyte. J. Appl. Phys..

[150] Wetjen M., Kim G.-T., Joost M., Winter M., Passerini S. (2013). Temperature dependence of electrochemical properties of cross-linked poly(ethylene oxide)–lithium bis(trifluoromethanesulfonyl)imide–N-butyl-N-methylpyrrolidinium bis(trifluoromethanesulfonyl)imide solid polymer electrolytes for lithium batteries. Electrochim. Acta.

[151] Joost M., Kunze M., Jeong S., Schönhoff M., Winter M., Passerini S. (2012). Ionic mobility in ternary polymer electrolytes for lithium-ion batteries. Electrochim. Acta.

[152] Girolamo D.D., Panero S., Navarra M.A., Hassoun J. (2016). Quaternary Polyethylene Oxide Electrolytes Containing Ionic Liquid for Lithium Polymer Battery. J. Electrochem. Soc..

[153] Derrien G., Hassoun J., Sacchetti S., Panero S. (2009). Nanocomposite PEO-based polymer electrolyte using a highly porous, super acid zirconia filler. Solid State Ionics.

[154] Kimura K., Matsumoto H., Hassoun J., Panero S., Scrosati B., Tominaga Y. (2015). A QuaternaryPoly(ethylene carbonate)-Lithium Bis(trifluoromethanesulfonyl)imide-Ionic Liquid-Silica Fiber Composite Polymer Electrolyte for Lithium Batteries. Electrochim. Acta.

[155] Leones R., Sabadini R.C., Esperança J.M.S.S., Pawlicka A., Silva M.M. (2017). Playing with ionic liquids to uncover novel polymer electrolytes. Solid State Ionics.

[156] Li M., Yang L., Fang S., Dong S., Hirano S.I., Tachibana K. (2012). Polymerized ionic liquids with guanidinium cations as host for gel polymer electrolytes in lithium metal batteries. Polym. Int..

[157] Li M., Wang L., Yang B., Du T., Zhang Y. (2014). Facile preparation of polymer electrolytes based on the polymerized ionic liquid poly((4-vinylbenzyl)trimethylammonium bis(trifluoromethanesulfonylimide)) for lithium secondary batteries. Electrochim. Acta.

[158] Wu F., Chen N., Chen R., Wang L., Li L. (2017). Organically modified silica-supported ionogels electrolyte for high temperature lithium-ion batteries. Nano Energy.

[159] Guan J., Li Y., Li J. (2017). Stretchable Ionic-Liquid-Based Gel Polymer Electrolytes for Lithium-Ion Batteries. Ind. Eng. Chem. Res..

[160] Tan G., Wu F., Zhan C., Wang J., Mu D., Lu J., Amine K. (2016). Solid-State Li-Ion Batteries Using Fast, Stable, Glassy Nanocomposite Electrolytes for Good Safety and Long Cycle-Life. Nano Lett..

[161] Lee A.S., Lee J.H., Hong S.M., Lee J.C., Hwang S.S., Koo C.M. (2016). Boronic ionogel electrolytes to improve lithium transport for Li-ion batteries. Electrochim. Acta.

[162] Song S., Yang S., Zheng F., Zeng K., Duong H.M., Savilov S.V., Aldoshin S.M., Hu N., Lu L. (2016). Poly(ethylene oxide)-Immobilized Ionogel with High Ionic Liquid Loading and Superior Ionic Conductivity. J. Electrochem. Soc..

[163] Deng M.-J., Chang J.-K., Wang C.-C., Chen K.-W., Lin C.-M., Tang M.-T., Chen J.-M., Lu K.-T., Simon P., Gogotsi Y. (2011). High-performance electrochemical pseudo-capacitor based on MnO2 nanowires/Ni foam as electrode with a novel Li-ion quasi-ionic liquid as electrolyte. Energy Environ. Sci..

[164] Shayeh J.S., Ehsani A., Ganjali M.R., Norouzi P., Jaleh B. (2015). Conductive polymer/reduced graphene oxide/Au nano particles as efficient composite materials in electrochemical supercapacitors. Appl. Surf. Sci..

[165] Lee K.K., Chin W.S., Sow C.H. (2014). Cobalt-based compounds and composites as electrode materials for high-performance electrochemical capacitors. J. Mater. Chem. A.

[166] Raccichini R., Varzi A., Passerini S., Scrosati B. (2015). The role of graphene for electrochemical energy storage. Nat. Mater..

[167] Baran D., Kirchartz T., Wheeler B.S., Dimitrov S., Abdelsamie M., Gorman J., Ashraf R.S., Holliday S., Wadsworth A., Gasparini N. (2016). Reduced voltage losses yield 10% efficient fullerene free organic solar cells with >1 V open circuit voltages. Energy Environ. Sci. Energy Environ. Sci..

[168] Banerjee A., Upadhyay K.K., Puthusseri D., Aravindan V., Madhavi S., Ogale S. (2014). MOF-derived crumpled-sheet-assembled perforated carbon cuboids as highly effective cathode active materials for ultra-high energy density Li-ion hybrid electrochemical capacitors (Li-HECs). Nanoscale.

[169] Xu L.-L., Guo M.-X., Liu S., Bian S.-W. (2015). Graphene/cotton composite fabrics as flexible electrode materials for electrochemical capacitors. RSC Adv..

[170] Chen J., Li C., Shi G. (2013). Graphene Materials for Electrochemical Capacitors. J. Phys. Chem. Lett..

[171] Yu Z., Tetard L., Zhai L., Thomas J. (2015). Supercapacitor electrode materials: nanostructures from 0 to 3 dimensions. Energy Environ. Sci..

[172] Krishnan S.G., Reddy M.V., Harilal M., Vidyadharan B., Misnon I.I., Rahim M.H.A., Ismail J., Jose R. (2015). Characterization of MgCo2O4 as an electrode for high performance supercapacitors. Electrochim. Acta.

[173] Wang L., Zhang X., Wang S., Li Y., Qian B., Jiang X., Yang G. (2014). Ultrasonic-assisted synthesis of amorphous Fe3O4 with a high specific surface area and improved capacitance for supercapacitor. Powder Technol..

[174] Van Aken K.L., Beidaghi M., Gogotsi Y. (2015). Formulation of Ionic-Liquid Electrolyte to Expand the Voltage Window of Supercapacitors. Angew. Chem. Int. Ed..

[175] Singh M.P., Singh R.K., Chandra S. (2014). Ionic liquids confined in porous matrices: Physicochemical properties and applications. Prog. Mater. Sci..

[176] Tarascon J.M. (2010). Key challenges in future Li-battery research. Philos. Trans. R. Soc. A Math. Phys. Eng. Sci..

[177] Dunn B., Kamath H., Tarascon J.-M. (2011). Electrical Energy Storage for the Grid: A Battery of Choices. Science.

[178] Leones R., Sabadini R.C., Esperança J.M.S.S., Pawlicka A., Silva M.M. (2017). Effect of storage time on the ionic conductivity of chitosan-solid polymer electrolytes incorporating cyano-based ionic liquids. Electrochim. Acta.

[179] Lee W.G., Cho W.J., Whang Y.H., Raj C.J., Kim B.C., Park J.H., Yu K.H. (2016). Feasible study of polypyrrole film in single and double cationic ionic liquids as novel electrolytes for energy storage applications. Synth. Met..

[180] Sheberla D., Bachman J.C., Elias J.S., Sun C.J., Shao-Horn Y., Dincǎ M. (2017). Conductive MOF electrodes for stable supercapacitors with high areal capacitance. Nat. Mater..

[181] Oschatz M., Boukhalfa S., Nickel W., Hofmann J.P., Fischer C., Yushin G., Kaskel S. (2018). Crucial Factors for the Application of Functional Nanoporous Carbon-Based Materials in Energy and Environmental Applications. J. Carbon Res..

[182] Watanabe M., Thomas M.L., Zhang S., Ueno K., Yasuda T., Dokko K. (2017). Application of Ionic Liquids to Energy Storage and Conversion Materials and Devices. Chem. Rev..

[183] Borenstein A., Hanna O., Attias R., Luski S., Brousse T., Aurbach D. (2017). Carbon-based composite materials for supercapacitor electrodes: a review. J. Mater. Chem. A.

[184] Zhang L., Tsay K., Bock C., Zhang J. (2016). Ionic liquids as electrolytes for non-aqueous solutions electrochemical supercapacitors in a temperature range of 20 °C–80 °C. J. Power Sources.

[185] Kazemiabnavi S., Zhang Z., Thornton K., Banerjee S. (2016). Electrochemical Stability Window of Imidazolium-Based Ionic Liquids as Electrolytes for Lithium Batteries. J. Phys. Chem. B.

[186] Ramu M., Chellan J.R., Goli N., Joaquim P., Cristobal V., Kim B.C. (2020). A Self-Branched Lamination of Hierarchical Patronite Nanoarchitectures on Carbon Fiber Cloth as Novel Electrode for Ionic Liquid Electrolyte-Based High Energy Density Supercapacitors. Adv. Funct. Mater..

[187] Kim C.H., Wee J.-H., Kim Y.A., Yang K.S., Yang C.-M. (2016). Tailoring the pore structure of carbon nanofibers for achieving ultrahigh-energy-density supercapacitors using ionic liquids as electrolytes. J. Mater. Chem. A.

[188] Tsai W.Y., Lin R., Murali S., Zhang L.L., McDonough J.K., Ruoff R.S., Taberna P.L., Gogotsi Y., Simon P. (2013). Outstanding performance of activated graphene based supercapacitors in ionic liquid electrolyte from −50 to 80 °C. Nano Energy.

[189] Kurig H., Vestli M., Tonurist K., Janes A., Lust E. (2012). Influence of Room Temperature Ionic Liquid Anion Chemical Composition and Electrical Charge Delocalization on the Supercapacitor Properties. J. Electrochem. Soc..

[190] Denshchikov K.K., Izmaylova M.Y., Zhuk A.Z., Vygodskii Y.S., Novikov V.T., Gerasimov A.F. (2010). 1-Methyl-3-butylimidazolium tetraflouroborate with activated carbon for electrochemical double layer supercapacitors. Electrochim. Acta.

[191] Shaikh J.S., Pawar R.C., Devan R.S., Ma Y.R., Salvi P.P., Kolekar S.S., Patil P.S. (2011). Synthesis and characterization of Ru doped CuO thin films for supercapacitor based on Bronsted acidic ionic liquid. Electrochim. Acta.

[192] Brandt A., Pires J., Anouti M., Balducci A. (2013). An investigation about the cycling stability of supercapacitors containing protic ionic liquids as electrolyte components. Electrochim. Acta.

[193] Maiti S., Pramanik A., Chattopadhyay S., De G., Mahanty S. (2016). Electrochemical energy storage in montmorillonite K10 clay based composite as supercapacitor using ionic liquid electrolyte. J. Colloid Interface Sci..

[194] Abdallah T., Lemordant D., Claude-Montigny B. (2012). Are room temperature ionic liquids able to improve the safety of supercapacitors organic electrolytes without degrading the performances?. J. Power Sources.

[195] Chen Y., Zhang X., Zhang D., Ma Y. (2012). High power density of graphene-based supercapacitors in ionic liquid electrolytes. Mater. Lett..

[196] Yang C.H., Huang P.L., Luo X.F., Wang C.H., Li C., Wu Y.H., Chang J.K. (2015). Holey Graphene Nanosheets with Surface Functional Groups as High-Performance Supercapacitors in Ionic-Liquid Electrolyte. ChemSusChem.

[197] Shao Q., Tang J., Lin Y., Li J., Qin F., Yuan J., Qin L.C. (2015). Carbon nanotube spaced graphene aerogels with enhanced capacitance in aqueous and ionic liquid electrolytes. J. Power Sources.

[198] Fuertes A.B., Sevilla M. (2015). High-surface area carbons from renewable sources with a bimodal micro-mesoporosity for high-performance ionic liquid-based supercapacitors. Carbon.

[199] Jha N., Ramesh P., Bekyarova E., Itkis M.E., Haddon R.C. (2012). High Energy Density Supercapacitor Based on a Hybrid Carbon Nanotube–Reduced Graphite Oxide Architecture. Adv. Energy Mater..

[200] Pinkert K., Oschatz M., Borchardt L., Klose M., Zier M., Nickel W., Giebeler L., Kaskel S., Eckert J. (2014). Role of Surface Functional Groups in Ordered Mesoporous Carbide-Derived Carbon/Ionic Liquid Electrolyte Double-Layer Capacitor Interfaces. ACS Appl. Mater. Interfaces.

[201] Lei Z., Liu Z., Wang H., Sun X., Lu L., Zhao X.S. (2013). A high-energy-density supercapacitor with graphene–CMK-5 as the electrode and ionic liquid as the electrolyte. J. Mater. Chem. A.

[202] Chang H.-H., Chang C.-K., Tsai Y.-C., Liao C.-S. (2012). Electrochemically synthesized graphene/polypyrrole composites and their use in supercapacitor. Carbon.

[203] Anouti M., Couadou E., Timperman L., Galiano H. (2012). Protic ionic liquid as electrolyte for high-densities electrochemical double layer capacitors with activated carbon electrode material. Electrochim. Acta.

[204] Kang Y.J., Chung H., Han C.-H., Kim W. (2012). All-solid-state flexible supercapacitors based on papers coated with carbon nanotubes and ionic-liquid-based gel electrolytes. Nanotechnology.

[205] Sangchoom W., Walsh D.A., Mokaya R. (2018). Valorization of lignin waste: high electrochemical capacitance of lignin-derived carbons in aqueous and ionic liquid electrolytes. J. Mater. Chem. A.

[206] Wang G., Ling Z., Li C., Dong Q., Qian B., Qiu J. (2013). Ionic liquid as template to synthesize carbon xerogels by coupling with KOH activation for supercapacitors. Electrochem. Commun..

[207] Pandey G.P., Rastogi A.C., Westgate C.R. (2014). All-solid-state supercapacitors with poly(3,4-ethylenedioxythiophene)-coated carbon fiber paper electrodes and ionic liquid gel polymer electrolyte. J. Power Sources.

[208] Feng L., Wang K., Zhang X., Sun X., Li C., Ge X., Ma Y. (2018). Flexible Solid-State Supercapacitors with Enhanced Performance from Hierarchically Graphene Nanocomposite Electrodes and Ionic Liquid Incorporated Gel Polymer Electrolyte. Adv. Funct. Mater..

[209] Xu L., Xia J., Xu H., Yin S., Wang K., Huang L. (2014). Reactable ionic liquid assisted solvothermal synthesis of graphite-like C3N4 hybridized α-Fe_2_O_3_ hollow microspheres with enhanced supercapacitive performance. J. Power Sources.

[210] Kim J., Kim S. (2014). Preparation and electrochemical property of ionic liquid-attached graphene nanosheets for an application of supercapacitor electrode. Electrochim. Acta.

[211] Li X., Liu Y., Guo W., Chen J., He W., Peng F. (2014). Synthesis of spherical PANI particles via chemical polymerization in ionic liquid for high-performance supercapacitors. Electrochim. Acta.

[212] Kühnel R.S., Balducci A. (2014). Comparison of the anodic behavior of aluminum current collectors in imide-based ionic liquids and consequences on the stability of high voltage supercapacitors. J. Power Sources.

[213] Huang P.L., Luo X.F., Peng Y.Y., Pu N.W., Ger M.D. (2015). Ionic Liquid Electrolytes with Various Constituent Ions for Graphene-based Supercapacitors. Electrochim. Acta.

[214] Tooming T., Thomberg T., Kurig H., Jänes A., Lust E. (2015). High power density supercapacitors based on the carbon dioxide activated d-glucose derived carbon electrodes and 1-ethyl-3-methylimidazolium tetrafluoroborate ionic liquid. J. Power Sources.

[215] Dubal D.P., Aradilla D., Bidan G., Gentile P., Schubert T.J.S., Wimberg J., Sadki S., Gomez-Romero P. (2015). 3D hierarchical assembly of ultrathin MnO2 nanoflakes on silicon nanowires for high performance micro-supercapacitors in Li- doped ionic liquid. Sci. Rep..

[216] Guo N., Li M., Wang Y., Sun X., Wang F., Yang R. (2016). Soybean Root-Derived Hierarchical Porous Carbon as Electrode Material for High-Performance Supercapacitors in Ionic Liquids. ACS Appl. Mater. Interfaces.

[217] Jin Z.Y., Lu A.H., Xu Y.Y., Zhang J.T., Li W.C. (2014). Ionic Liquid-Assisted Synthesis of Microporous Carbon Nanosheets for Use in High Rate and Long Cycle Life Supercapacitors. Adv. Mater..

[218] Wei L., Sevilla M., Fuertes A.B., Mokaya R., Yushin G. (2012). Polypyrrole-Derived Activated Carbons for High-Performance Electrical Double-Layer Capacitors with Ionic Liquid Electrolyte. Adv. Funct. Mater..

[219] Wang X., Li Y., Lou F., Buan M.E.M., Sheridan E., Chen D. (2017). Enhancing capacitance of supercapacitor with both organic electrolyte and ionic liquid electrolyte on a biomass-derived carbon. RSC. Adv..

[220] Kim T.Y., Lee H.W., Stoller M., Dreyer D.R., Bielawski C.W., Ruoff R.S., Suh K.S. (2010). High-Performance Supercapacitors Based on Poly(ionic liquid)-Modified Graphene Electrodes. ACS Nano.

[221] Guo D.-C., Mi J., Hao G.-P., Dong W., Xiong G., Li W.-C., Lu A.-H. (2013). A facile approach for the synthesis of monolithic hierarchical porous carbons–high performance materials for amine based CO_2_ capture and supercapacitor electrode. Energy Environ. Sci..

[222] Fu C., Kuang Y., Huang Z., Wang X., Yin Y., Chen J., Zhou H. (2011). Supercapacitor based on graphene and ionic liquid electrolyte. J. Solid State Electrochem..

[223] Forse A.C., Griffin J.M., Merlet C., Bayley P.M., Wang H., Simon P., Grey C.P. (2015). NMR Study of Ion Dynamics and Charge Storage in Ionic Liquid Supercapacitors. J. Am. Chem. Soc..

[224] Vu A., Li X., Phillips J., Han A., Smyrl W.H., Bühlmann P., Stein A. (2013). Three-Dimensionally Ordered Mesoporous (3DOm) Carbon Materials as Electrodes for Electrochemical Double-Layer Capacitors with Ionic Liquid Electrolytes. Chem. Mater..

[225] Sathish M., Mitani S., Tomai T., Honma I. (2014). Supercritical fluid assisted synthesis of N-doped graphene nanosheets and their capacitance behavior in ionic liquid and aqueous electrolytes. J. Mater. Chem. A.

[226] Brandt A., Ramirez-Castro C., Anouti M., Balducci A. (2013). An investigation about the use of mixtures of sulfonium-based ionic liquids and propylene carbonate as electrolytes for supercapacitors. J. Mater. Chem. A.

[227] Zhang X., Zhao D., Zhao Y., Tang P., Shen Y., Xu C., Li H., Xiao Y. (2013). High performance asymmetric supercapacitor based on MnO2 electrode in ionic liquid electrolyte. J. Mater. Chem. A.

[228] Tooming T., Thomberg T., Siinor L., Tonurist K., Janes A., Lust E. (2013). High Capacitance Supercapacitor Based on Mixed Room Temperature Ionic Liquids Containing Specifically Adsorbed Iodide Anions. J. Electrochem. Soc..

[229] Timperman L., Beguin F., Frackowiak E., Anouti M. (2013). Comparative Study of Two Protic Ionic Liquids as Electrolyte for Electrical Double-Layer Capacitors. J. Electrochem. Soc..

[230] Thangavel R., Kannan A.G., Ponraj R., Thangavel V., Kim D.-W., Lee Y.-S. (2018). High-energy green supercapacitor driven by ionic liquid electrolytes as an ultra-high stable next-generation energy storage device. J. Power Sources.

[231] Bettini L.G., Galluzzi M., Podestà A., Milani P., Piseri P. (2013). Planar thin film supercapacitor based on cluster-assembled nanostructured carbon and ionic liquid electrolyte. Carbon N. Y..

[232] Xie H.J., Gélinas B., Rochefort D. (2016). Redox-active electrolyte supercapacitors using electroactive ionic liquids. Electrochem. Commun..

[233] Lin Z., Taberna P.L., Simon P. (2016). Graphene-Based Supercapacitors Using Eutectic Ionic Liquid Mixture Electrolyte. Electrochim. Acta.

[234] Ujjain S.K., Sahu V., Sharma R.K., Singh G. (2015). High performance, All solid state, flexible Supercapacitor based on Ionic liquid functionalized Graphene. Electrochim. Acta.

[235] Tamilarasan P., Ramaprabhu S. (2014). Stretchable supercapacitors based on highly stretchable ionic liquid incorporated polymer electrolyte. Mater. Chem. Phys..

[236] Lee J., Kim W., Kim W. (2014). Stretchable Carbon Nanotube/Ion–Gel Supercapacitors with High Durability Realized through Interfacial Microroughness. ACS Appl. Mater. Interfaces.

[237] Liew C.W., Ramesh S., Arof A.K. (2014). Good prospect of ionic liquid based-poly(vinyl alcohol) polymer electrolytes for supercapacitors with excellent electrical, electrochemical and thermal properties. Int. J. Hydrog. Energy.

[238] Pandey G.P., Liu T., Hancock C., Li Y., Sun X.S., Li J. (2016). Thermostable gel polymer electrolyte based on succinonitrile and ionic liquid for high-performance solid-state supercapacitors. J. Power Sources.

[239] Yamagata M., Soeda K., Ikebe S., Yamazaki S., Ishikawa M. (2013). Chitosan-based gel electrolyte containing an ionic liquid for high-performance nonaqueous supercapacitors. Electrochim. Acta.

[240] Shi M.J., Kou S.Z., Shen B.S., Lang J.W., Yang Z., Yan X.B. (2014). Improving the performance of all-solid-state supercapacitors by modifying ionic liquid gel electrolytes with graphene nanosheets prepared by arc-discharge. Chin. Chem. Lett..

[241] Liew C., Ramesh S., Arof A.K. (2014). Characterization of ionic liquid added poly(vinyl alcohol)-based proton conducting polymer electrolytes and electrochemical studies on the supercapacitors. Int. J. Hydrog. Energy.

[242] Singh P.K., Sabin K.C., Chen X. (2016). Ionic liquid–solid polymer electrolyte blends for supercapacitor applications. Polym. Bull..

[243] Nawaz A., Sharif R., Rhee H.W., Singh P.K. (2016). Efficient dye sensitized solar cell and supercapacitor using 1-ethyl 3-methyl imidazolium dicyanamide incorporated PVDF–HFP polymer matrix. J. Ind. Eng. Chem..

[244] Yang X., Zhang F., Zhang L., Zhang T., Huang Y., Chen Y. (2013). A High-Performance Graphene Oxide-Doped Ion Gel as Gel Polymer Electrolyte for All-Solid-State Supercapacitor Applications. Adv. Funct. Mater..

[245] Pandey G.P., Hashmi S.A., Kumar Y. (2010). Performance Studies of Activated Charcoal Based Electrical Double Layer Capacitors with Ionic Liquid Gel Polymer Electrolytes. Energy Fuels.

[246] Pandey G.P., Hashmi S.A. (2013). Ionic liquid 1-ethyl-3-methylimidazolium tetracyanoborate-based gel polymer electrolyte for electrochemical capacitors. J. Mater. Chem. A.

[247] Pandey G.P., Hashmi S.A., Kumar Y. (2010). Multiwalled Carbon Nanotube Electrodes for Electrical Double Layer Capacitors with Ionic Liquid Based Gel Polymer Electrolytes. J. Electrochem. Soc..

